# An Open‐Label Dose‐Finding Study of Allopurinol to Target Defined Reduction in Urate Levels in Hemodialysis Patients

**DOI:** 10.1002/jcph.939

**Published:** 2017-06-09

**Authors:** Elaine Rutherford, Graham Stewart, J. Graeme Houston, Alan G. Jardine, Patrick B. Mark, Allan D. Struthers

**Affiliations:** ^1^ Institute of Cardiovascular & Medical Sciences, BHF Cardiovascular Research Centre, University of Glasgow Glasgow UK; ^2^ Renal & Transplant Unit Queen Elizabeth University Hospital Glasgow UK; ^3^ Renal Unit Ninewells Hospital & Medical School Dundee UK; ^4^ Division of Molecular & Clinical Medicine School of Medicine Ninewells Hospital and Medical School Dundee UK

**Keywords:** allopurinol, clinical trial, dosing, hemodialysis, urate

Allopurinol is a xanthine oxidase inhibitor mainly used to reduce circulating urate (also known as uric acid) levels. This helps to prevent recurrent urate crystal deposition in the form of gout.^1^ Gout is relatively common in patients with renal failure. Although routine dose reduction of allopurinol is recommended in the hemodialysis (HD) population this is not based on evidence from robust clinical trials.^2^ Guidelines generally recommend aiming to reduce urate levels by 50% to protect against future gout episodes.^3^ This study aimed to determine the optimal dose of allopurinol to achieve this in the HD population.

Determining an optimal dose of allopurinol to use in the HD population will first help to guide clinicians in their treatment of their HD patients who develop gout. Second, patients with end‐stage renal disease are at greatly increased risk of cardiovascular disease and death.^4^ Urate levels are known to be higher in the chronic kidney disease (CKD) population than in the general population.^5^ We know from large observational studies that increased urate levels are strongly associated with an increased cardiovascular risk, in both the general and CKD populations.^6^, ^7^, ^8^, ^9^

In addition to its urate‐lowering abilities, allopurinol itself has some specific properties that make it a potentially attractive drug to use in the HD population. Allopurinol has been shown to improve endothelial function in several population groups—including CKD patients—who are prone to endothelial dysfunction.^10^, ^11^, ^12^ In a large observational study the use of allopurinol was associated with reduced cardiovascular and all‐cause mortality in patients undergoing HD who had no history of cardiovascular disease.^13^ In addition to this, allopurinol has also been demonstrated to regress left ventricular hypertrophy (LVH) in CKD, diabetic patients and in those with ischemic heart disease.^10^, ^11^, ^14^ Given that reduction in left ventricular mass is a common therapeutic target in the HD population,^15^, ^16^, ^17^, ^18^ it is a natural question whether allopurinol might also regress LVH in this population. Until now this had not been investigated in a robust prospective clinical trial.

We therefore designed a clinical trial to investigate this, the ALTERED (Does *AL*lopurinol regress lef*T* ventricular hypertrophy in *E*nd stage *RE*nal *D*isease) study. This study looked to address this question through a multicenter randomized, placebo‐controlled, double‐blind trial of allopurinol. The primary outcome of the ALTERED study is change in left ventricular mass measured by cardiac magnetic resonance imaging at follow‐up from baseline after 1 year of therapy. For the reasons described above, defining the optimal dose of allopurinol to use in this study was a priority. The first stage of the ALTERED study was therefore an open‐label dose‐escalation study to determine the optimum dose of allopurinol to use in the prospective blinded study. The results of this dose‐finding study are presented in this article.

## Methods

This study was approved by the Medicines and Healthcare Products Regulatory Agency (United Kingdom; EudraCT number 2013‐001436‐22) and by the East of Scotland Ethics Committee (13/ES/0051). All participants gave full written and informed consent. The study was registered online with clinicaltrials.gov (trial number: NCT01951404). Protocol version 1.1, dated April 11, 2013, was in use during this dose‐finding study (see Supplementary Information for full protocol).

The primary outcome of this dose‐finding study was to determine which dose of active allopurinol reduced serum urate by 41% in the HD population. A secondary outcome of this study, in addition to the main study, was to help to determine the safety and tolerability of allopurinol in this population group.

Patients were eligible for this open‐label dose‐finding study if they were aged 18 to 80 years, had end‐stage renal disease (CKD stage 5), and had been on HD for at least 3 months.

Patients for the dose‐finding study were recruited as representative of those to be studied in the larger HD clinical trial. Exclusion criteria were: known heart failure or left ventricular ejection fraction < 45%; already had gout or taking allopurinol; severe hepatic disease; on azathioprine, 6‐mercaptopurine, theophylline, or warfarin; malignancy or other life‐threatening diseases; pregnant or lactating women; any contraindication to magnetic resonance imaging; with a planned (relative) kidney transplant; younger than 18 years or older than 80 years; above‐ankle amputees; patients who had participated in any other clinical trial within the previous 30 days; patients who were unable to give informed consent; and any other reason considered by a study physician to make someone inappropriate for study inclusion.

The study protocol specified that 10 patients would have a screening visit at which serum urate would be checked. Blood was sampled from the venous port of the HD circuit prior to commencement of the HD session. Thereafter, patients would receive open‐label 100 mg of allopurinol after each dialysis session for 3 sessions. Serum urate was then checked at the commencement of the fourth dialysis session. Thereafter, the allopurinol dose was increased to 200 mg and the same procedure repeated. The next step was 250 mg and then 300 and 350 mg (maximum dose). In keeping with published recommendations to ensure safe prescribing of allopurinol in end‐stage renal disease,^19^ the dose escalation was planned to stop in each patient, when serum urate had fallen by 50% from the screening visit urate in that patient.

Safety blood tests, vital signs, adverse events, and medication records were reviewed at each study visit. Using the collected pilot data, we planned to calculate which of the above doses of allopurinol (given at the end of each dialysis session) produced on average a 41% fall in serum urate. We planned to use this dose for the prospective blinded trial.

All patients received their usual dialysis care throughout their participation in the study. Dialysis modalities are shown in Table 1.

**Table 1:** Baseline Patient Characteristics

Patient Parameter^a^	Value
Male (%)	90
Age (years)	53 ± 9.4
White (%)	100
Body mass index (kg/m^2^)	25.0 (IQR 21.8–26.9)
Diabetes (%)	30
Hypertension (%)	80
Ischemic heart disease (%)	10
Dialysis mode: post‐HDF (%)	80
Hemodialysis (%)	20
Urea reduction ratio (%)	72 ± 7.0
Ultrafiltration volume (L)	1.5 ± 1.2
Predialysis systolic BP (mm Hg)	152 ± 22.0
Predialysis diastolic BP (mm Hg)	69.8 ± 17.9

For baseline patient characteristics, mean ± standard deviation are presented for normally distributed data and median (interquartile range) is presented for nonnormally distributed data or percentage of whole cohort where relevant.

BP, blood pressure; HDF, hemodiafiltration; IQR, interquartile range.

a Parameters given are values at baseline visit.

### Statistical Methods

All data were analyzed using SPSS version 22. Continuous data are presented as mean ± standard deviation if parametric or median (interquartile range) if nonnormally distributed; categorical data are presented as a percentage. After testing for normal distribution, a repeated‐measures analysis of variance with a Greenhouse‐Geisser correction was used to determine any statistically significant difference in urate at each dose of allopurinol. A Bonferroni correction was used to adjust for within‐subject baseline urate in our analysis. Correlations between continuous indices were assessed using Pearson and Spearman correlation coefficients for parametric and nonparametric data, respectively. *P* < .05 was considered statistically significant.

## Results

Eleven patients, all of whom were receiving regular HD at Ninewells Hospital, Dundee, Scotland, UK, consented to participate in the study. All patients commenced the study during October 2013. On screening, 1 participant was ineligible; therefore, 10 participants, 9 men and 1 woman, proceeded into the open‐label study. Full participant demographics and baseline parameters of the included participants are shown in Table 1.

The mean urate on study commencement was 6.3 ± 1.1 mg/dL (normal laboratory reference range: males, 3.4–7.2 mg/dL; females, 2.4–6.1 mg/dL). Baseline urate was negatively correlated with age (*r* = ‐0.766; *P* = .01). There was no correlation of baseline urate with blood pressure, ultrafiltration volume, urea reduction ratio, or body mass index.

No patient reached a urate reduction of 50% on any dose of allopurinol. The greatest individual percentage reduction in urate by any patient was 45.4%, in a single patient while taking allopurinol 350 mg. This was achieved in the patient with the highest starting urate (baseline urate, 8.3 mg/dL), the only patient with a baseline urate outside the normal range.

**Figure 1:**
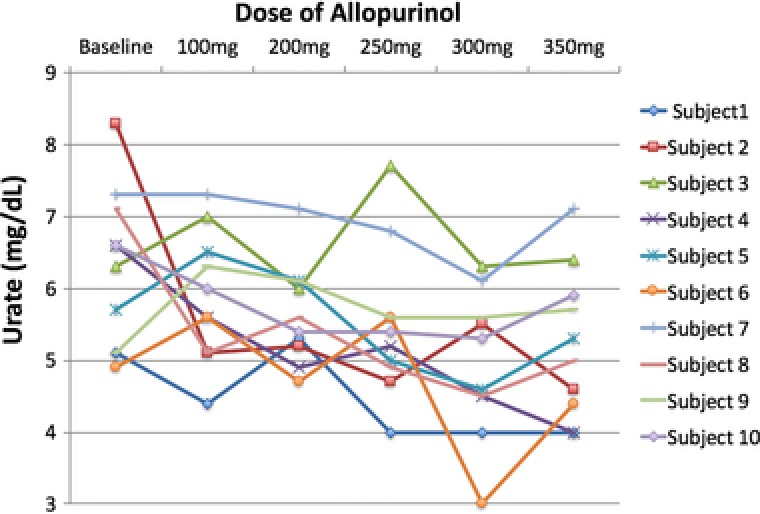
Plot of individual urates at each dose of allopurinol.

**Figure 2:**
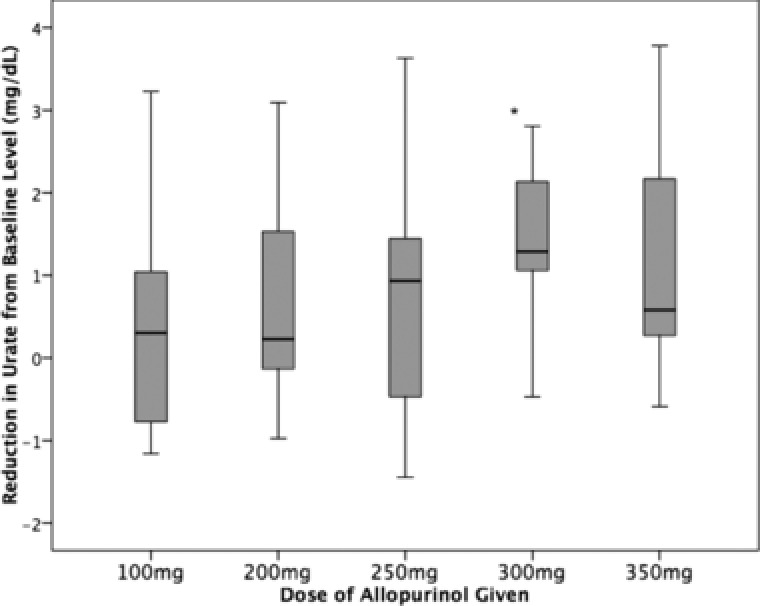
Box plot of reduction in urate from baseline with each dose of allopurinol, with 95% confidence intervals. A significant mean reduction in urate was seen with the 300‐mg dose of allopurinol. *P < .05.

Overall, only allopurinol 300 mg achieved a statistically significant reduction in predialysis serum urate from baseline (mean urate at baseline, 6.3 ± 1.1 mg/dL; visit 6 [allopurinol 300 mg], 4.9 ± 1.0 mg/dL; *P* = .04; Table 2). Figure 1 shows a plot of urates for each patient at each dose of allopurinol. The greatest mean reduction in urate was achieved with the 300‐mg dose of allopurinol (see Figure 2).

**Table 2:** Mean Serum Urate Values at Each Dose of Allopurinol

Visit	Mean Urate ± Standard Deviation (mg/dL)	*P* a
Baseline	6.3 ± 1.1	N/A
After 100 mg	5.9 ± 0.9	1.0
After 200 mg	5.6 ± 0.7	1.0
After 250 mg	5.5 ± 1.1	1.0
After 300 mg	4.9 ± 1.0	0.04
After 350 mg	5.2 ± 1.0	0.5

a Adjusted for baseline urate using a Bonferroni correction.

There were 22 adverse events during the course of the study. All were minor in nature and typical of normal events seen in dialysis patients. Two episodes of nausea and a single episode of loose stool were the only events that could possibly have been attributed to allopurinol—these 3 episodes were all self‐limiting. There were no reported skin rashes. There were no statistically significant changes in white cell count, hemoglobin, liver function tests, phosphate, or potassium from the baseline visit to the end of the study (Table 3). The cumulative dose of allopurinol and number of doses of allopurinol that each patient had been exposed to at each visit are also shown in Table 3.

**Table 3:** Summary of Exposure to Allopurinol and Safety Blood Tests at Each Study Visit

Variable	Laboratory Reference Range	Baseline	Visit 3	Visit 4	Visit 5	Visit 6	Visit 7
Expected cumulative allopurinol exposure per patient (mg)	Not applicable	0	300	900	1650	2550	3600
Expected number of doses of allopurinol per patient	Not applicable	0	3	6	9	12	15
White cell count (cells/mm^3^)	4000–11 000	7630 ± 2894	7350 ± 2690	7750 ± 2577	7840 ± 2768	8250(IQR 7050–8900)	7820 ± 3111
Hemoglobin (g/dL)	13–18	12.0 ± 1.2	11.2 ± 0.9	11.4 ± 1.0	11.1 ± 0.9	11.1 ± 0.6	10.9 ± 0.5
Albumin (g/dL)	3.5–5.0	3.3 ± 0.4	3.4 ± 0.4	3.4 ± 0.4	3.3 ± 0.4	3.3 ± 0.5	3.3 ± 0.5
Bilirubin (mg/dL)	0–1.2	0.3 (IQR 0.2–0.4)	0.3 (IQR 0.2–0.4)	0.2 (IQR 0.2–0.3)	0.3 ± 0.2	0.3 ± 0.1	0.3 ± 0.2
Alkaline phosphatase (U/L)	30–130	117 ± 64	107 ± 50	102 ± 42	99 ± 39	104 ± 45	86 (IQR 74–93)
Alanine aminotransferase (U/mL)	5–55	18.2 ± 5.7	17.8 ± 6.1	16.9 ± 5.2	17.8 ± 6.1	18.1 ± 6.1	17.9 ± 7.7
Phosphate (mg/dL)	2.5–4.6	5.6 ± 0.6	5.9 ± 1.2	5.6 ± 0.9	5.6 ± 0.9	5.6 ± 1.2	5.6 ± 0.9
Potassium (mmol/L)	3.5–5.3	5.3 ± 0.6	5.2 ± 0.5	5.2 ± 0.7	5.2 ± 0.5	5.4 ± 0.6	5.3 ± 0.5

Data is presented as mean ± standard deviation or median (IQR 1–3). IQR, interquartile range.

## Discussion

### Study Rationale

Since its discovery more than 50 years ago, allopurinol has been the mainstay therapy for prevention of recurrent gout.^1^ It is also indicated for the prophylaxis of hyperuricemia associated with malignancy or with the treatment of malignancy.^20^ Allopurinol is further utilized in the management of renal stone disease (both calcium oxalate stones and uric acid stones).^20^

More recently there has been emerging interest in the potential utility of allopurinol to reduce cardiovascular disease risk.^21^ We have known for some time that there appears to be a link between urate level, heart disease, and mortality.^8^ We also know that higher urate levels are often found in disease states such as chronic kidney disease and diabetes, which are themselves associated with an increased cardiovascular risk.^22^ However, reduction in urate alone is not enough to reduce cardiovascular risk in at‐risk populations.^23^ In addition, the association between urate level and mortality is slightly more complex in the HD population than in other populations. The majority of studies suggest a J‐shaped mortality relationship with urate exists, with both low and high levels of urate associated with an increased mortality risk.^24^, ^25^, ^26^, ^27^ This is likely to reflect that the lowest urate levels are found in frailer and less well‐nourished HD patients. This J‐shaped relationship does not detract from the fact that for most HD patients, reducing urate levels is a potential therapeutic target to try to reduce an elevated cardiovascular risk.

Allopurinol has been shown to regress left ventricular mass in ischemic heart disease, in type 2 diabetes, and in early chronic kidney disease.^10^, ^11^, ^14^ The reasons allopurinol might regress left ventricular mass are severalfold. First, allopurinol profoundly reduces oxidative stress. Oxidative stress is a well‐known important driver of left ventricular hypertrophy.^28^ Allopurinol has also been shown to significantly improve arterial endothelial function^11^, ^29^ and can lead to improved arterial compliance and reduced ventricular afterload.^14^, ^30^ Reduced ventricular afterload because of better arterial compliance can regress left ventricular hypertrophy.^11^, ^14^ As left ventricular hypertrophy is associated with increased mortality,^31^ regression of left ventricular mass by allopurinol may be associated with the same increased survival as seen in other clinical trials in which regression of left ventricular mass has been achieved.^32^ However, a large clinical trial of allopurinol versus placebo focusing on major adverse cardiovascular events in a vulnerable group would be required to show the ability of allopurinol to achieve this.

In the general population, a treat‐to‐target approach can be used to determine urate dosing with doses of up to 900 mg per day being licensed to achieve a significant reduction in urate levels.^3^ For patients with end‐stage renal disease requiring HD, there are no data to guide management. The maximum recommended dose in the United Kingdom is 300 mg after HD (ie, every second day).^2^ However, until now, little was known about the overall effect such a dose had on circulating urate levels. Our dose‐finding study has shown that allopurinol at doses up to 350 mg does not achieve large reductions in urate. This is likely to be due to dialysis‐related factors.

First, urate is a small compound and is therefore, like urea, largely removed by dialysis at each HD session. This means that after each dialysis session, patients will start with a low circulating serum urate. In the interdialytic gap, urate will reaccumulate, as it will not be excreted as normal via the renal tract. This intermittent removal of urate is likely to explain why this group of patients largely had a predialysis urate within the normal range, whereas patients in a chronic kidney disease allopurinol study (who were not receiving dialysis) had slightly more elevated baseline urates.^11^ The urate levels seen in that previous chronic kidney disease study are in keeping with patients with chronic kidney disease (CKD) being commonly found to have higher levels of circulating urate.^5^

Another dialysis‐specific factor in this study is that allopurinol itself is also removed by dialysis. This means that if a single allopurinol tablet was missed at the preceding dialysis session to the study visit dialysis session, then there would be effectively no allopurinol in the patient's system. Although compliance was very good in this study, on 2 confirmed occasions when an allopurinol tablet was missed, urate was not reduced at the next session.

### Why Aim for a Fixed Urate Reduction?

We chose a target reduction in urate of 41%, as we considered this a reasonable and safe target for urate lowering for both clinical and research purposes. In previous work, in the CKD population, a urate reduction of 41% was associated with a significant regression in left ventricular mass.^11^ The regression in left ventricular mass was independent of urate reduction, which supportes the theory that the novel actions of allopurinol other than urate lowering are important. This means it is possible to have some confidence that the potential benefits of allopurinol might still be seen in the dialysis population, even though their urate levels were not dramatically reduced.

It is worth noting that allopurinol is generally used with caution in the CKD population because of concerns over an increased incidence of skin reactions including the very rare but serious allopurinol hypersensitivity syndrome.^33^ Accordingly, dose reduction is recommended and normal in the renal population.^2^ In keeping with published recommendations to ensure safe prescribing of allopurinol in end‐stage renal disease,^19^ the dose escalation was therefore planned to stop in each patient when serum urate had fallen by 50% from the screening visit urate in that patient. In this study, we saw no serious adverse events, and the only 3 adverse events that could have possibly been related to allopurinol were separate self‐limiting episodes of nausea and diarrhea. Reassuringly there were also no adverse changes in safety blood tests that could be attributed to allopurinol. Therefore, although it was very small, this study lends some support for allopurinol at doses up to 300 and 350 mg being well tolerated in this population.

This study has several limitations. First, this was a very small single‐center study with a relatively short duration of investigation. The population was not racially diverse and was 90% male. Given the small numbers in this study, urate levels could have also been affected by other factors outside the control of the study team (for example, a shortened dialysis session because of vascular access issues). Nevertheless, similarly sized studies have been used by the Food and Drug Administration for the licensing of other drugs in end‐stage renal disease, including rivaroxiban and dabigatran.^34^, ^35^ The urate reduction by allopurinol demonstrated in this study was less than originally anticipated. However, despite this, the investigators believe that this dose‐finding study was sufficient to allow an informed decision about which dose of allopurinol to safely use for the main ALTERED trial. The results of the main trial may provide further clinically relevant information that will assist nephrologists when prescribing allopurinol for clinical purposes.

## Conclusion

We sought to determine which dose of allopurinol, up to 350 mg, would significantly reduce urate in a group of 10 dialysis patients. No dose of allopurinol reduced urate by 50%, and this is likely to be because of dialysis‐related factors. However, in this small study, all doses of allopurinol were well tolerated, and 300 mg was associated with the most significant drop in urate levels. The dose of 300 mg was chosen as the dose of allopurinol to be used in the double‐blind, placebo‐controlled ALTERED study, which seeks to answer the question, does allopurinol regress left ventricular mass in end‐stage renal disease? This study suggests that 300 mg may be a reasonable dose of allopurinol to use in dialysis patients if a significant reduction in urate is sought for clinical purposes. Lower doses of allopurinol are unlikely to have a significant effect on urate levels. The larger double‐blind study of allopurinol 300 mg against placebo will add further useful information about the safety and tolerability of such a dose in dialysis populations.

## Supporting information

Additional Supporting Information may be found in the online version of this article at the publisher's website.
