# Supplementary material for: An Open‐Label Dose‐Finding Study of Allopurinol to Target Defined Reduction in Urate Levels in Hemodialysis Patients
Source: J Clin Pharmacol. 2017 Jun 9;57(11):1409–14. doi: 10.1002/jcph.939 (PMC5655768; doi:10.1002/jcph.939)
Supplement: Supplementary file 1 — Additional Supporting Information may be found in the online version of this article at the publisher's website. [file JCPH-57-1409-s001.docx]

**Study Protocol**

Does **AL**lopurinol regress lef**T** ventricular hypertrophy in **E**nd stage **RE**nal Disease: The **ALTERED** study

| Sponsor | University of Dundee-NHS Tayside |
| --- | --- |
| Funder | British Heart Foundation Ref: 29743 |
| Chief Investigator | Professor Allan D Struthers |
| Principal Investigator &  Clinical Research Fellow | Dr Elaine Rutherford |
| EudraCT Number | 2013-001436-22 |
| CTA Number | TBA |
| REC Number | 13/ES/0051 |
| ISRCTN Number | TBA |
| Version Number and Date | Version 1.1 11th April 2013 |
| Sponsor R&D Number | 2012CV07 |

#### contents

[contents 2](#_Toc353865670)

[protocol approval 4](#_Toc353865671)

[list of abbreviations 5](#_Toc353865672)

[summary 6](#_Toc353865673)

[1.1 BACKGROUND 7](#_Toc353865674)

[1.2 RATIONALE FOR STUDY 9](#_Toc353865675)

[2 OBJECTIVES 10](#_Toc353865676)

[2.1 STUDY OBJECTIVEs 10](#_Toc353865677)

[2.1.1 Primary Objective 10](#_Toc353865678)

[2.1.2 Secondary Objectives 10](#_Toc353865679)

[2.2 OUTCOMES 11](#_Toc353865680)

[2.2.1 Primary Outcomes 11](#_Toc353865681)

[2.2.2 Secondary Outcomes 11](#_Toc353865682)

[3 STUDY DESIGN 12](#_Toc353865683)

[3.1.1 Participant follow-up schedule of the Altered Trial (Phase 1: Dose escalation) 12](#_Toc353865684)

[3.1.2 Participant follow-up schedule of the Phase 2 ALTERED Trial 12](#_Toc353865685)

[4 STUDY POPULATION 16](#_Toc353865686)

[4.1 NUMBER OF PARTICIPANTS 16](#_Toc353865687)

[4.2 INCLUSION CRITERIA 16](#_Toc353865688)

[4.3 EXCLUSION CRITERIA 16](#_Toc353865689)

[5 PARTICIPANT SELECTION AND ENROLMENT 17](#_Toc353865690)

[5.1 IDENTIFYING PARTICIPANTS 17](#_Toc353865691)

[5.2 CONSENTING PARTICIPANTS 17](#_Toc353865692)

[5.3 SCREENING FOR ELIGIBILITY 17](#_Toc353865693)

[5.4 INELIGIBLE AND NON-RECRUITED PARTICIPANTS 17](#_Toc353865694)

[5.5 RANDOMISATION 17](#_Toc353865695)

[5.5.1 Randomisation 17](#_Toc353865696)

[5.5.2 Treatment Allocation 18](#_Toc353865697)

[5.5.3 Emergency Unblinding Procedures 18](#_Toc353865698)

[5.5.4 Withdrawal procedures 18](#_Toc353865699)

[6 INVESTIGATIONAL MEDICINAL PRODUCT 19](#_Toc353865700)

[6.1 STUDY DRUG 19](#_Toc353865701)

[6.1.1 Study Drug Identification 19](#_Toc353865702)

[6.1.2 Study Drug Manufacturer 19](#_Toc353865703)

[6.1.3 Marketing Authorisation Holder 19](#_Toc353865704)

[6.1.4 Labelling and Packaging 19](#_Toc353865705)

[6.1.5 Storage 19](#_Toc353865706)

[6.1.6 Summary of Product Characteristics 19](#_Toc353865707)

[6.1.7 Accountability procedures 19](#_Toc353865708)

[6.2 STUDY COMPARATOR 19](#_Toc353865709)

[6.2.1 Comparator Identification 19](#_Toc353865710)

[6.2.2 Comparator Manufacturer 19](#_Toc353865711)

[6.2.3 Labelling and Packaging 20](#_Toc353865712)

[6.2.4 Storage 20](#_Toc353865713)

[6.3 DOSING REGIME 20](#_Toc353865714)

[6.4 DOSE CHANGES 20](#_Toc353865715)

[6.5 PARTICIPANT COMPLIANCE 20](#_Toc353865716)

[6.6 OVERDOSE 20](#_Toc353865717)

[6.7 OTHER MEDICATIONS 21](#_Toc353865718)

[6.7.1 Permitted Medications 21](#_Toc353865719)

[6.7.2 Prohibited Medications 21](#_Toc353865720)

[6.7.3 Concurrent Medications 21](#_Toc353865721)

[7 STUDY ASSESSMENTS 21](#_Toc353865722)

[7.1 STUDY VISITS 21](#_Toc353865723)

[7.2 SAFETY ASSESSMENTS 25](#_Toc353865724)

[8 DATA COLLECTION & MANAGEMENT 25](#_Toc353865725)

[8.1 Data Collection 25](#_Toc353865726)

[8.2 Data Management System 25](#_Toc353865727)

[9 STATISTICS AND DATA ANALYSIS 25](#_Toc353865728)

[9.1 SAMPLE SIZE CALCULATION 25](#_Toc353865729)

[9.2 PROPOSED ANALYSES 25](#_Toc353865730)

[9.3 MISSING DATA 26](#_Toc353865731)

[10 ADVERSE EVENTS 26](#_Toc353865732)

[10.1 DEFINITIONS 26](#_Toc353865733)

[10.2 DETECTING AEs AND SAEs 26](#_Toc353865734)

[10.3 RECORDING AEs AND SAEs 27](#_Toc353865735)

[10.4 EVALUATION OF AEs AND SAEs 27](#_Toc353865736)

[10.4.1 Assessment of Seriousness 27](#_Toc353865737)

[10.4.2 Assessment of Causality 27](#_Toc353865738)

[10.4.3 Assessment of Severity 28](#_Toc353865739)

[10.4.4 Assessment of Expectedness 28](#_Toc353865740)

[10.5 REPORTING OF SAEs/SARs/SUSARs 28](#_Toc353865741)

[10.6 REGULATORY REPORTING REQUIREMENTS 28](#_Toc353865742)

[10.7 URGENT SAFETY MEASURES 29](#_Toc353865743)

[11 PREGNANCY 29](#_Toc353865744)

[12 TRIAL MANAGEMENT AND OVERSIGHT ARRANGEMENTS 29](#_Toc353865745)

[12.1 TRIAL MANAGEMENT GROUP 29](#_Toc353865746)

[12.2 TRIAL MANAGEMENT 29](#_Toc353865747)

[12.3 TRIAL STEERING COMMITTEE 30](#_Toc353865748)

[12.4 DATA MONITORING COMMITTEE 30](#_Toc353865749)

[12.5 INSPECTION OF RECORDS 30](#_Toc353865750)

[12.6 RISK ASSESSMENT 30](#_Toc353865751)

[12.7 STUDY MONITORING 30](#_Toc353865752)

[12.7.1 Potential Risks 30](#_Toc353865753)

[12.7.2 Minimising Risk 30](#_Toc353865754)

[13 GOOD CLINICAL PRACTICE 30](#_Toc353865755)

[13.1 ETHICAL CONDUCT OF THE STUDY 30](#_Toc353865756)

[13.2 REGULATORY COMPLIANCE OF THE STUDY 31](#_Toc353865757)

[13.3 CI RESPONSIBILITIES 31](#_Toc353865758)

[13.3.1 Informed Consent 31](#_Toc353865759)

[13.3.2 Study Staff 31](#_Toc353865760)

[13.3.3 Data Recording 31](#_Toc353865761)

[13.3.4 CI Documentation 31](#_Toc353865762)

[13.3.5 GCP Training 32](#_Toc353865763)

[13.3.6 Confidentiality 32](#_Toc353865764)

[13.3.7 Data Protection 32](#_Toc353865765)

[14 STUDY CONDUCT RESPONSIBILITIES 32](#_Toc353865766)

[14.1 PROTOCOL AMENDMENTS 32](#_Toc353865767)

[14.2 PROTOCOL VIOLATIONS AND DEVIATIONS 32](#_Toc353865768)

[14.3 STUDY RECORD RETENTION 33](#_Toc353865769)

[14.4 END OF STUDY 33](#_Toc353865770)

[14.5 CONTINUATION OF DRUG FOLLOWING THE END OF STUDY 33](#_Toc353865771)

[15 REPORTING, PUBLICATIONS AND NOTIFICATION OF RESULTS 33](#_Toc353865772)

[15.1 AUTHORSHIP POLICY 33](#_Toc353865773)

[15.2 PUBLICATION 33](#_Toc353865774)

[15.3 PEER REVIEW 33](#_Toc353865775)

[16 REFERENCES 34](#_Toc353865776)

[APPENDIX 1: Trial Steering Committee 37](#_Toc353865777)

[APPENDIX 2 PHASE 1 Dose ESCALATION study (OPEN LABEL) 38](#_Toc353865778)

[Appendix 3: Main PHASE 2 Randomised Controlled Trial 39](#_Toc353865779)

[APPENDIX 4 CONSORT 40](#_Toc353865780)

#### protocol approval

**Does Allopurinol regress left ventricular hypertrophy in end stage renal disease? The ALTERED study**

EudraCT number : 2013-001436-22

**Signatures**

**By signing this document I am confirming that I have read, understood and approve the protocol for the above study.**

| Professor Allan D Struthers |  |  |  |
| --- | --- | --- | --- |
| Chief Investigator | Signature |  | Date |
|  |  |  |  |
|  |  |  |  |
|  |  |  |  |
| Dr Elaine Rutherford |  |  |  |
| Research Fellow/  Principal Investigator | Signature |  | Date |
|  |  |  |  |
|  |  |  |  |
|  |  |  |  |
|  |  |  |  |
|  |  |  |  |
|  |  |  |  |
|  |  |  |  |
|  |  |  |  |
|  |  |  |  |

#### list of abbreviations

| AF | Atrial Fibrillation |
| --- | --- |
| AIx | Augmentation Index |
| AR | Adverse reaction |
| BNP | Brain Naturetic Peptide |
| BP | Blood Pressure |
| CI | Chief Investigator |
| CMR (I) | Cardiac Magnetic Resonance (Imaging) |
| CKD | Chronic Kidney Disease |
| CTIMP | Clinical Trial of Investigational Medicinal Product |
| CV | Cardiovascular |
| DBP | Diastolic Blood Pressure |
| DSUR | Development Safety Update Reporting |
| ECG | Electrocardiogram |
| EDV | End diastolic volume |
| ESRD | End Stage Renal Disease |
| FMD | Flow Mediated Dilatation |
| GCP | Good Clinical Practice |
| GTN | Glyceryl Trinitrate |
| LV | Left Ventricular |
| LVH | Left Ventricular Hypertrophy |
| MR (I) | Magnetic Resonance (Imaging) |
| NRES | National Research Ethics Service |
| PI | Principal Investigator |
| PIS | Participant Information Sheet |
| PWA | Pulse Wave Analysis |
| PVD | Peripheral Vascular Disease |
| SBP | Systolic Blood Pressure |
| SmPC | Summary of Product Characteristics |
| SAR | Serious Adverse Reaction |
| TCTU | Tayside Clinical Trials Unit |
| TMF | Trial Master File |
| XO | Xanthine Oxidase |

#### summary

***Lay***

Kidney patients on dialysis commonly die because of heart disease. One of the biggest problems in their hearts is that the muscle wall of the heart thickens. This makes it less efficient. We found in patients with mild kidney disease that a drug normally used to treat gout (allopurinol) had the remarkable side effect of being able to reduce this thickening of their heart wall. In this new study we aim to find out if this benefit of allopurinol also occurs in severe kidney patients i.e. those on regular dialysis. We also are trying to figure out the best dose of allopurinol to use. To do this we are planning a study where we will recruit patients with kidney disease who are on dialysis. The 1^st^ phase of the trial will be to determine the best dose of allopurinol to use and the second phase will be to do a clinical trial where patients will be randomly allocated to either this optimum dose of allopurinol or a dummy medication (placebo) and will receive one year of treatment. They will have a special scan of the heart using an MRI machine to measure the extent of thickening of their heart muscle before they start on treatment and will have a further MRI scan when their one year treatment finishes.

Phase 1- the dose finding study, will involve 10 patients who will have between 3 and 7 visits to the hospital scheduled around 4 to 17 dialysis sessions. The later study will involve up to 76 patients who will be asked to attend the hospital up to 8 times over a 13 month period.

***Professional Summary***

Cardiovascular (CV) events and deaths are extremely common in renal dialysis patients. A large contributor to this is their high incidence of left ventricular hypertrophy (LVH). In earlier work, we found that allopurinol was able to regress LVH in patients with mild renal disease. We now wish to see if this occurs also in patients with more severe renal disease who are undergoing regular haemodialysis. We will perform an initial dose ranging study to select the dose of allopurinol which we will use. We will then randomise 76 dialysis patients into a randomised double blind placebo controlled trial of allopurinol. The primary endpoint will be MRI measured LV mass but we will also get added value by measuring MR spectroscopy for myocardial energetics as well as endothelial/vascular function by several techniques: flow mediated dilatation, pulse wave analysis and aortic compliance (by MR). We will establish whether allopurinol reduces LVH and improves endothelial dysfunction in dialysis patients. If so, this would justify seeking funding for a large mega trial to see if allopurinol reduces CV events and deaths in dialysis patients.

**INTRODUCTION**

## BACKGROUND

The annual mortality of patients with end stage renal disease (ESRD) approaches 10% per year, which is 10-30 times higher than the general population.[[1](#_ENREF_1)] Almost half of these deaths are due to cardiovascular (CV) events which are attributed to a mixture of coronary disease and left ventricular abnormalities, especially left ventricular hypertrophy (LVH). In fact among those commencing dialysis, 74% have LVH on echocardiography.[[2](#_ENREF_2)] This agrees with more recent data where LVH was present in 78% of those with chronic kidney disease (CKD) stages 3-5.[[3](#_ENREF_3)] The reasons why LVH is so common in CKD are probably multiple but a strong contributor is thought to be oxidative stress which is known to mediate LVH and to be greatly increased in renal disease.[[4](#_ENREF_4), [5](#_ENREF_5)] The potential importance of oxidative stress in CKD can also be illustrated by the fact that antioxidant vitamins have failed to reduce CV events in all studies, with the singular exception of the SPACE trial in ESRD patients where Vitamin E produced a 54% reduction (p=0.014) in CV events (MI, ischaemic stroke, unstable angina and PVD).[[6](#_ENREF_6)] Although this finding could have been chance, the SPACE trial result accords well with the fact that CKD patients are known to have much more oxidative stress than any other patient group.

Interest in echo LVH as a strong independent cardiovascular risk factor began with the Framingham study where it was found that, after age, LVH was the strongest predictor there is of CV events and death.[[7](#_ENREF_7)] Even in patients undergoing coronary angiography, echo LVH is a stronger predictor of death (RR 2.4) than either multi-vessel coronary disease or LV systolic dysfunction.[[8](#_ENREF_8)] In renal patients, LVH is equally bad. For example in patients commencing renal replacement therapy (RRT), the independent risk ratio for LVH was 2.9 for all-cause mortality and 2.7 for CV mortality and this was after adjustment for age, known coronary disease, systolic BP, diabetes and treatment (dialysis or transplantation).[[9](#_ENREF_9)] The reason why LVH carries such a bad CV prognosis is probably because it leads to so many different types of adverse CV sequelae. For example, LVH is intrinsically arrhythmogenic which lead to a doubling of sudden cardiac death.[[10](#_ENREF_10)] Furthermore LVH leads to diastolic heart failure.[[11](#_ENREF_11), [12](#_ENREF_12)] Both arrhythmogenicity and heart failure are very important in ESRD since sudden arrhythmic death and LV failure are commoner modes of death than coronary atherosclerosis. In addition the stiff left ventricle of LVH also causes left atrial dilation which in turn leads to atrial fibrillation and cardioembolic strokes.[[13](#_ENREF_13)] Lastly LVH also reduces coronary perfusion reserve.

The question naturally arises whether regressing LVH would actually lead to a reduction in CV events. Here the data are clear that regressing LVH unequivocally reduces subsequent CV events, independently of changes in blood pressure. In Framingham, LVH regression conferred a 54% reduction in CV events in men and a 44% reduction in women.[[14](#_ENREF_14)] The LIFE study was the biggest study (n=9193) to address this question and blood pressure was eliminated as a factor here since it was the same in both groups. In LIFE, LVH regression independently reduced sudden death by 30%, it reduced new onset AF by 12% and it reduced new heart failure by 36%.[[15-17](#_ENREF_15)] From the above and much other work, LVH regression appears to be a therapeutic target which reliably indicates that a reduction in CV events/mortality will follow if LVH regresses. Indeed, Mancini et al [[18](#_ENREF_18)] recently wrote in Circulation that “we now have the ultimate documentation for LVH that its reversal has independent prognosis value, independent of BP”. Schillaci et al [[19](#_ENREF_19)] also wrote that “LVH stands out as the only marker whose regression has been unequivocally associated with a better prognosis”. LVH regression also appears beneficial in ESRD patients. London et al [[20](#_ENREF_20)] found that CV free survival at 70 months was 90% in those with >10% regression in LV mass, compared to 50% in those with <10% regression in LV mass.

Therefore in ESRD, LVH is extremely common, it confers a poor prognosis and its regression is almost certain to reduce CV events/death. Hence new treatments to regress LVH in ESRD need to be developed which should first be assessed by whether they really do regress LVH. Such studies would be considered “proof of concept” studies enabling only new therapies which are positive in such studies to be taken forward to large trials with CV events as their endpoint. In this way, such “proof of concept” studies enable us to select only the most promising new treatments to go forward to expensive mega trials. Clearly we do not live in an ideal world where we could afford expensive mega trials for all potential new treatments and using reliable surrogates like LVH regression is necessary to select the most promising options from among all the possible options.

The current established way to regress LVH is to achieve target blood pressure. Patients with ESRD are aggressively treated to achieve target BP already but, as mentioned above, LVH often persists despite these aggressive attempts. When renal function allows it, the control of BP in ESRD often includes the use of ACE inhibitors and/or ARBs, as they are thought to be a little better than other anti-hypertensives at regressing LVH.

We recently found a completely novel way to regress LVH in CKD patients on top of conventional treatment. We found that allopurinol (300mg/day) significantly regressed LVH (as measured by MRI) in patients with mild (stage 3) CKD. [[21](#_ENREF_21)] In fact, in as yet unpublished work, we have found allopurinol (600mg) to also regress LVH in patients with chronic stable angina (p=0.007). In both studies, including the CKD patients, allopurinol was well tolerated and renal function stable. In fact, one paper suggests that allopurinol improves renal function in CKD.[[22](#_ENREF_22)] The effect that we saw on LVH in two studies in man agrees with two animal studies where allopurinol also regressed LVH.[[23](#_ENREF_23), [24](#_ENREF_24)] There are probably two inter-related reasons why allopurinol regressed LVH in these 4 studies (2 human, 2 animal). The first is that allopurinol profoundly reduces oxidative stress and oxidative stress is a key driver of LVH. [[4](#_ENREF_4), [25](#_ENREF_25)] The second is that allopurinol markedly improves arterial endothelial dysfunction and this improved arterial compliance then reduces LV afterload which is another major driver of LVH i.e. even without changing BP itself, reduced LV afterload due to better arterial compliance can regress LVH.[[21](#_ENREF_21), [25](#_ENREF_25)] In support of this is our observation of a significant correlation between allopurinol induced changes in endothelial function and allopurinol induced changes in LV mass in mild CKD.[[21](#_ENREF_21)]

Having shown clearly that allopurinol can regress LVH in mild (stage 3) CKD patients, we feel that the next stage is to see if this beneficial effect occurs also in patients with severe CKD i.e. patients with ESRD. After all, it looks from large clinical trials that lowering cholesterol is beneficial in mild CKD but not in the dialysis population, which shows that separate studies are always necessary in these two populations and that one cannot extrapolate from one population to the other.[[26](#_ENREF_26), [27](#_ENREF_27)] This result with cholesterol lowering is probably because coronary atherosclerosis is the main culprit in mild CKD whereas the main culprits in ESRD are LVH driven events such as arrhythmias and heart failure. This is further justification for using LVH regression as the main endpoint when seeking new treatments to reduce the high incidence of CV events/mortality in ESRD.

Our proposed study in ESRD would not only provide “proof of concept” evidence enabling this new therapy to move forward to be tested in an endpoint trial in ESRD but it would also act as important confirmation of the whole concept that allopurinol can regress LVH in CKD. After all no one is going to fund an endpoint mega trial in ESRD without this (necessary) piece of evidence in this exact cohort of patients. In doing this trial in ESRD we shall see if allopurinol improves another important surrogate i.e. endothelial dysfunction. Since allopurinol has always improved endothelial function in all previous patient studies, it seems likely that it will do so again in ESRD and this study will be an opportunity to acquire these key data which will help make the case for a mega trial in ESRD. It will also produce pilot data that allopurinol is likely to be tolerated and safe in ESRD which will also be essential, as a preliminary to an ESRD mega trial. What will assist also in eventually making the case for this future mega trial is a small (n=113) study where allopurinol reduced CV events by 71% although admittedly this was in patients with mild CKD.[[22](#_ENREF_22)] Since CV events are commoner in ESRD than in mild CKD, an endpoint trial in ESRD would also be less expensive. Indeed, it is traditional to do such mega trials first in severe disease and only later in milder disease, because CV event rates are higher in severe disease.

## RATIONALE FOR STUDY

The wider aim is to answer three key questions that need to be answered before any large trial can be designed to assess the effect of allopurinol on CV events and mortality in ESRD. More specifically, we plan to answer the following key questions in this project.

Firstly, what is the optimum minimum dose of allopurinol in order to realise a 41% reduction in serum urate levels in ten patients with ESRD?

Secondly, once optimal minimum allopurinol result is determined, what is the effect of allopurinol on LVH in patients with ESRD?

Thirdly, we will for the first time ever establish whether allopurinol improves cardiac energetics in ESRD in man.

This information is essential to inform on the future design of a large trial to investigate whether allopurinol really can reduce CV events and mortality in patients with ESRD.

**EXPERIMENTAL DESIGN**

To answer the two main study questions the study will be split into two parts; an open label dose escalation study followed by the main ALTERED trial using the optimum dose of allopurinol as decided by the preliminary study.

**Phase 1 Dose Escalation Study**

A preliminary study will be undertaken to see which dose of active allopurinol reduces plasma urate by the same margin (41%) as seen in our CKD stage 3 study where allopurinol did regress LVH.[[21](#_ENREF_21)]

Ten patients with end stage renal disease (CKD stage 5) who have been on haemodialysis for at least 3 months will initially receive open label 100mg allopurinol **after each** dialysis session for 3 sessions. Plasma urate will be checked at the commencement of the fourth dialysis session. Thereafter the allopurinol dose will be increased to 200mg and the same procedure repeated. The next step will be 250mg and then 300mg and 350mg (maximum dose). The dose escalation will stop in each patient when plasma urate has fallen by 50% in that patient which is in keeping with published recommendations to ensure safe prescribing of allopurinol in ESRD. [[28](#_ENREF_28)] From these pilot data, we will calculate which of the above doses of allopurinol (given at the end of each dialysis session) produces on average a 41% fall in plasma urate. This will be the chosen dose for the main trial.

**Phase 2: Main ALTERED Trial**

The main trial will be a 12 month, placebo-controlled, randomised, double-blind, parallel-group study. After baseline assessments and investigations, patients will be then randomly assigned to receive an allopurinol capsule (100mg, start dose then increased to maximum dose decided from the dose ranging preliminary phase of the trial) or placebo capsule for 2 weeks of dialysis sessions (typically 6 dialysis sessions).

Randomisation will be done by Tayside Pharmaceuticals and will be stratified by site to ensure balance. Each dose will be given at the end of each dialysis session.

If this 100mg start dose is tolerated, this would be increased at weekly intervals to the allopurinol dose chosen by the pilot study. Baseline blood samples will be taken for full blood count, renal function, liver function, random blood glucose, haemoglobin A1C, lipids, calcium and phosphate, and these would be repeated at 6, 9 and 12 months. However, urate levels will be stored and measured at the end of the study in order to ensure blinding throughout. Subjects will be followed at baseline, week 2, week 6, month 5, month 9 and 12 with close monitoring of their full blood count and renal function. Office BP will be measured thrice at each visit and the average of the second and third reading will be recorded and also pre and post dialysis. BP is recorded as part of standard care and will be available for analysis. 24 hour BP will also be measured at baseline and at 12 months.

**Study Population**

We will recruit 76 patients with end stage renal disease (CKD stage 5 eGFR <15ml/min /1.73m^2^) who have been on haemodialysis for at least 3 months. They will also have to have echocardiographic LVH according to the new ASE (American Society of Echocardiography) criteria of LVM ≥115g/m^2^ for men and >95g/m^2^ for women. Patients will be excluded if any of the following criteria are present: already on allopurinol, active gout, known heart failure, LVEF <45% on echo, (as this would indicate the whole extra disease of heart failure) severe hepatic disease, current immunosuppressive therapy, azathioprine, warfarin, mercaptopurine, theophylline, malignancy or other life threatening diseases, pregnant or lactating women or any contraindication to MRI (claustrophobia, metal implants). To enhance study completion, we will exclude those with a planned (relative) transplant, those over 80 years and amputees: the latter two categories have a particularly high mortality rate. It is standard in studies such as these to use echo to screen but to use MRI to detect treatment effects. This is because the whole world literature on what LVH cut-offs give a poor prognosis are based on echo but MRI is essential to detect treatment effects due to its excellent reproducibility.

**EXPECTED VALUE**

This study will establish whether allopurinol really does reduce LVH and improve endothelial/vascular function in dialysis patients. If it did, then this could fully justify a large mega trial to see if allopurinol reduces the extremely high rate of CV events and deaths in such patients. Such a large mega trial in ESRD could be relatively good value overall since CV events/deaths are so high in ESRD.

The results on endothelial/vascular function will be particularly robust as we are measuring it by three different techniques i.e. FMD, PWA and MR. The chance of positive results on LVH and endothelial function is very high since we found allopurinol to have such positive effects in mild renal disease.

Added value will however be gained in this study from three other previously unstudied issues. Firstly from our initial dose ranging, we will establish a reasonable dose of allopurinol for dialysis patients. Secondly we will get preliminary data on the safety and tolerability of allopurinol in dialysis patients. Thirdly, we will for the first time ever establish whether allopurinol improves cardiac energetics in ESRD in man. A very new publication has just found that allopurinol does improve cardiac energetics (by MRI) in heart failure.[[31](#_ENREF_31)]

# OBJECTIVES

## STUDY OBJECTIVEs

### Primary Objective

The primary objective will be to see if Allopurinol can regress left ventricular hypertrophy (as measured by MRI) in patients with ESRD.

### Secondary Objectives

- To decide on optimum dosing regime of allopurinol in ESRD from pilot study.
- To assess effect of allopurinol on endothelial function as measured by FMD and PWA.
- To assess safety of allopurinol in ESRD.

## OUTCOMES

### Primary Outcomes

The primary outcome is to determine if allopurinol, induces a change in Left ventricular Mass Index in patients with ESRD when compared to placebo.

### Secondary Outcomes

Secondary outcomes are:

- To determine if there is a change in LV end systolic volume, LV end diastolic volume or LV ejection factor with allopurinol in ESRD patients compared with placebo.
- To determine if there is a difference in endothelial function with allopurinol compared with placebo, measured by FMD and PWA
- To determine if there are changes in inflammatory blood markers, in ESRD with allopurinol compared with placebo.
- To assess, if there are changes in BP control as measured by clinic BP and 24hr BP monitoring with allopurinol compared with placebo

# STUDY DESIGN

### Participant follow-up schedule of the Altered Trial (Phase 1: Dose escalation)

### 3.1.2 Participant follow-up schedule of the Phase 2 ALTERED Trial

Excluded:

- Did not meet inclusion criteria

Allocated to Allopurinol Dose escalation study.

Start on 100mg allopurinol increase after every 3^rd^ dialysis session

Review after every 3^rd^ dialysis session for urate level and safety

**Treatment Allocation**

Open Label Phase 1 Study (n=10 completed)

if >50% decrease from baseline in urate level **stop** in trial or continue until 350mg allopurinol max dose

**Analysis**

**Follow-Up**

**Enrolment**

Excluded:

- Declined to participate

Opportunistic recruitment at dialysis sessions or via dialysis databases at NHS Tayside or NHS Greater Glasgow and Clyde

Invited to participate:

- Meets inclusion criteria

Informed Consent

Opportunistic recruitment at dialysis sessions or via dialysis databases at NHS Tayside or NHS Greater Glasgow and Clyde

Excluded:

- Did not meet inclusion criteria

**Reviews**

Safety review at 2 weeks, weekly up to 6 weeks. 6 month, 9 month and final 12 month review

**Reviews**

Safety review at 2 weeks, weekly up to 6 weeks. 6 month, 9 month and final 12 month review

Randomisation (n=76)

**Analysis**

**Follow-Up**

**Treatment Allocation**

Excluded:

- Declined to participate

Invited to participate:

- Meets inclusion criteria

Informed Consent

**Phase 1 (Dose Escalation) Study** (see study schedule page 35)

**Primary Outcome**

- Change in LV Mass Index

**Secondary Outcomes**

- Change in LV end systolic volume, LV end diastolic volume, or LVEF.
- Changes in FMD or PWA
- Changes in Blood Markers
- Changes in BP control

**Primary Outcome**

- Change in LV Mass Index

**Secondary Outcomes**

- Change in LV end systolic volume, LV end diastolic volume, or LVEF.
- Changes in FMD or PWA
- Changes in Blood Markers
- Changes in BP control

**Group B** - Allocated to placebo group (n= 38)

Start on 2 week (6 dialysis session) loading dose then increase weekly up to dose decided in pilot study

**Group A** - Allocated to Allopurinol group (n=38)

Start on 2 week(6 dialysis session) loading dose then increase weekly up to dose decided in pilot study

**Enrolment**

The Phase 1 dose escalation study is an open label study undertaken to see which dose of active allopurinol reduces plasma urate by the same margin (41%) as seen in our previous CKD stage 3 study where allopurinol did regress LVH.[[21](#_ENREF_21)]

Ten patients with CKD stage 5 will initially receive 100 mg allopurinol daily after each dialysis session for a total of 3 dialysis sessions. Plasma urate will be checked at the commencement of the fourth dialysis session. Thereafter the allopurinol dose will be increased to 200mg and the same procedure repeated. The next step will be 250mg and then 300mg and 350mg (max).

The dose escalation will **stop** in each patient when plasma urate has fallen by >50% in that patient which is in keeping with published recommendations to ensure safe prescribing of allopurinol in ESRD. [[28](#_ENREF_28)]

From these pilot data, we will calculate which of the above doses of allopurinol (given at the end of each dialysis session) produces on average a 41% fall in plasma urate. This will be the chosen dose for the ALTERED trial.

There will be a pause between the two phases of the study to calculate the optimum dose and prepare IMPs required for the main phase 2 trial.

**Phase 2: ALTERED Trial**

The ALTERED trial is a randomised, double blinded, placebo controlled multi-centre study conducted in NHS Tayside & NHS Greater Glasgow & Clyde to compare allopurinol (dose to be confirmed from dose escalation study noted above) at either, 100mg, 200mg, 250mg, 300mg or 350mg to placebo.

Patients will be enrolled in this trial for a period of between 12 to 13 months.

At screening visit an initial history and clinical examination will be performed. Participants will then undergo an echocardiogram unless they have had an echo in the previous 2 years. Eligible participants will have to have echo defined LVH as per American Society of Echocardiography criteria of LVM ≥115g/m^2^ for men and >95g/m^2^ for women.

Should the participant have echo defined LVH, they will return for a Cardiac Magnetic Resonance Imaging (MRI) scan at a later date in the screening period, prior to their baseline (Randomisation) visit.

They will also have bloods taken for safety analysis, have a 12 lead ECG done and have 24 hour BP monitoring and vital signs recorded.

Once the patient is known to be eligible they will return - for the first randomisation, dosing visit at any time up to four weeks after screening. At this randomisation visit post dialysis session, eligible participants will be randomly assigned to either placebo or allopurinol 100mg.

They will continue on allopurinol/placebo 100mg for 2 weeks, with dosing after each dialysis session only. They can have FMD, PWV and PWA measurements taken. All participants will be offered the opportunity to opt in or out of the FMD, PWV and PWA measurements which are secondary outcome measures only.

If study drugs are tolerated, the dose would be increased at weekly intervals after the 200mg dose to the allopurinol dose chosen by the dose escalation study, if greater than 200mg. Baseline blood samples will be taken for full blood count, renal function, liver function, random blood glucose, haemoglobin A1C, lipids, calcium and phosphate, and these will be repeated at 6, 9 and 12 months, bar lipids which are tested at baseline and final visit only. However, urate levels will be stored and measured at the end of the study in order to ensure blinding throughout. Subjects will be followed at baseline, week 2, week 6, month 6, month 9 and 12 with close monitoring of their full blood count and renal function. Research bloods for BNP measurement and inflammatory markers will be taken at baseline, and at months 6, 9 and the final 12 month study visit. Office BP will be measured thrice at each visit and the average of the second and third reading will be recorded. Furthermore pre and post dialysis. BP is recorded as part of standard care and will be available for analysis. 24 hour BP will also be measured at baseline and at 12 months.

Patients will continue with all their usual medication, which will remain unchanged throughout unless clinically indicated.

**CMRI Methods**

The MRIs will always be done on a post-dialysis day. Baseline and repeat CMR examinations in screening (between 1-4 weeks before randomisation) and after the final 12 month (+/- 2 weeks) visit will be performed on similar 3T Magnetom scanners (Siemens, Erlangen, Germany) using dedicated phase array cardiac and phosphorous spectroscopy coils. Serial contiguous short-axis cines will be acquired from the vertical long axis and horizontal long axis of the left ventricle (electrocardiogram gated, steady-state free precession imaging [true fast imaging with steady-state precession], with the short axis imaging parameters being a repetition time of 2.5ms, echo time of 1.1ms, flip angle of 60°, and slice thickness 6mm). Analysis will be performed offline (Argus Software, Siemens) by a single blinded observer for the assessment of ventricular volumes (EDV, ESV, stroke volume), EF, and left ventricular mass. This single observer will analyse scans from both sites. The reproducibility of the left ventricular mass assessment using MRI will be derived for this observer. A test-retest intraobserver coefficient of variation of 2.0% is usual in our past MRI studies. We will also assess reproducibility between each site, although the same observer will assess all scans at both sites. We will take the opportunity to measure two other MRI parameters of interest which are easily obtained with little extra scan time: Phosphorous -31 MR spectroscopy and aortic compliance by methods already published by us.[[29](#_ENREF_29), [30](#_ENREF_30)] Both will give significant added value.

MR spectroscopy will see whether allopurinol increases high energy phosphates in ESRD (measured by PCr:ATP) which it is likely to do since it is known to increase ATP levels in vitro and it is now known to improve cardiac energetics (MRI measured) in human heart failure.[[31](#_ENREF_31), [32](#_ENREF_32)] We will measure high energy phosphates (HEP), comparing Phosphocreatine : Beta adenosine triphosphate (PCr:ATP) ratio from the long axis views of the left ventricle.[[29](#_ENREF_29)] In addition we will use MRI to assess aortic vascular compliance and pulse wave velocity [[30](#_ENREF_30)] using cine transverse thoracic aortic gated steady state free precession acquisitions which will complement well the other peripheral vascular measures of endothelial/vascular function being assessed (see below).

**FMD**

FMD will be offered to all participants in the main trial, but may be declined without affecting their participation in the main trial.

FMD on the brachial artery will be performed in the non-fistula arm on three visits (baseline, month 9 and month 12) using a Philips iE33 ultrasound machine (Phillips Medical Systems, United Kingdom) according to the guide-lines set by the International Brachial Artery Reactivity Task Force.[[33](#_ENREF_33)] The brachial artery will be longitudinally imaged above the elbow using an 11.3-MHz probe. The image will be recorded for 2 minutes, followed by induction of forearm ischaemia by inflating a cuff below the elbow to 200 mmHg (or 50 mmHg above SBP, whichever is higher) for 5 minutes and deflating rapidly. The resulting reactive hyperaemia will be recorded for a further 2 minutes. After a rest period of 10 minutes, the procedure will be repeated, with 0.4mg of glyceryl trinitrate being administered sublingually to determine endothelium-independent dilation. FMD will be expressed as per cent change in diameter relative to the baseline diameter at rest. Analysis of all FMDs will be performed on the Brachial Analyser version 5.0 software (Medical Imaging Applications, LLC) by a single trained investigator (the clinical research fellow) to avoid inter-observer variability. This investigator will be blind to allocated treatments. Our intra-observer coefficient of variation for FMD is usually 5.2%. Two factors should ensure reliable results despite there being two sites : each patient will always be studied on the same equipment and the same individual (the research fellow) will do all the tests and interpret them (blindly) at both sites. For further reassurance, blinded ultrasound scans will be re-analysed to ensure good reproducibility at each site and between sites.

**Applanation Tonometry**

PWA will be offered to all participants in the main trial, but may be declined without affecting their participation in the main trial.

Pulse wave analysis (PWA) and PWV will be determined in the non-fistula arm by recording the radial waveforms and radial-carotid waveforms, respectively, at three visits (baseline, month 9 and month 12) using the Sphygmocor system. The central AIx will be corrected to a heart rate of 75 beats/min. A single trained investigator (the clinical research fellow) who is blind to the allocated treatment will perform the PWA and PWV at both sites. We will also use bio-impedance at the same time to measure total body water in MRIs done at Glasgow sites.[[21](#_ENREF_21)] . This will complement the end diastolic volume data from the MRI at both sites. In our index study, EDV fell but we did not know if this was due to afterload reduction or to a global fall in body water.[[21](#_ENREF_21)] Bio-impedance will help determine this.

**Research Blood tests**

Research bloods will be taken at baseline and at the end of treatment phase for BNP and inflammatory markers.

**Adverse events**

Adverse events will be sought by symptom enquiry at each visit and by monitoring of routine blood samples.

# STUDY POPULATION

## NUMBER OF PARTICIPANTS

For the Phase 1 Dose escalation study 10 eligible patients will be recruited from the dialysis sessions at any of the three NHS Tayside locations (Ninewells Hospital, Arbroath Royal Infirmary and Perth Royal Infirmary) or at six locations in Greater Glasgow & Clyde (Western Infirmary, Victoria Infirmary, Stobhill Hospital, Royal Alexandra Hospital, Inverclyde Royal, & Vale of Leven) by the clinical research fellow, who is a nephrology registrar.

For the Phase 2 ALTERED Trial 76 eligible patients will be recruited from the dialysis clinics at the two health boards.

It is anticipated that it will take three months to recruit and complete the Phase 1 dose escalation study and one year to recruit 76 eligible patients with approx. 114 being consented in the Phase 2 trial. We anticipate a screen failure rate of 25% and a dropout rate of 10% after recruitment.

## INCLUSION CRITERIA

They will be eligible if they

- are aged 18 to 80 years
- end stage renal disease (CKD stage 5 eGFR <15ml/min /1.73m^2^)
- been on haemodialysis for at least 3 months.
- For main study also require echocardiographic LVH according to the ASE (American Society of Echocardiography) criteria of LVM ≥115g/m^2^ for men and >95g/m^2^ for women.

## EXCLUSION CRITERIA

Exclusion criteria are

- Known heart failure
- Left Ventricular Ejection Fraction <45%,
- already had gout or on allopurinol,
- severe hepatic disease
- or on azathioprine, 6 mercaptopurine, theophylline or warfarin.
- malignancy or other life threatening diseases,
- pregnant or lactating women
- any contraindication to MRI (claustrophobia, metal implants).
- with a planned (relative) kidney transplant,
- Aged than 18 or over 80 years
- Above ankle amputees
- Patients who have participated in any other clinical trial within the previous 30 days will be excluded.
- Patients who are unable to give informed consent will also be excluded from this trial.
- Any other considered by a study physician to be inappropriate for inclusion

# PARTICIPANT SELECTION AND ENROLMENT

**(See CONSORT Diagram Appendix 4)**

## IDENTIFYING PARTICIPANTS

Participants will be recruited from current attendees at the dialysis clinics at Ninewells Hospital, Arbroath Royal Infirmary and Perth Royal Infirmary, and at six sites in Greater Glasgow & Clyde (Western General Infirmary, Victoria Infirmary, Stobhill Hospital, Royal Alexandra Hospital, Inverclyde Royal & Vale of Leven)

The research fellow or trial manager/research nurse will screen both the clinic lists and medical notes to identify suitable people (see below).

## CONSENTING PARTICIPANTS

Approaching potential participants and then obtaining consent will be carried out by the research fellow or trial manager/research nurse. The fellow or trial manager will approach suitable participants either at dialysis sessions or by letter sent from their renal consultant. Participants will be provided with a full participant information sheet (PIS). All participants will be given at least 24 hours to consider their response, which will initially be given either orally at the clinic or in writing by returning an expression of interest in a stamped addressed envelope. A screening visit will then be arranged where participants will be able to ask any questions about the study. If they decide to participate, informed consent will be taken and eligibility by all the criteria confirmed.

## SCREENING FOR ELIGIBILITY

Participants must have documented ESRD and be attending haemodialysis sessions Participants will be consented prior to screening and a screening log will be maintained.

## INELIGIBLE AND NON-RECRUITED PARTICIPANTS

The reason(s) for ineligibility will be explained to participants and any questions they have will be answered. They will be thanked for their participation in screening and any relevant information from this will be added to their hospital notes and be communicated to their GP and nephrologist where the patient consents for this to happen.

## RANDOMISATION

### Randomisation

There is no randomisation in the phase 1 dose escalation study. It is an open label study.

**For the main Phase 2 ALTERED trial:**

Participants will be allowed to continue all their usual medication throughout.

After successful screening for eligibility and safety, participants will be randomised to either allopurinol or placebo in a double blind fashion. Participants and their bloods (U&Es, LFTs, FBC) will be monitored as per study schedule and medication stopped or reduced in dose if concerns arise. If study drug dose is reduced, they will remain in the study. If study drug needs to be stopped, they will remain in the study in order to do an “intention to treat” analysis.

Double blind medication (allopurinol or placebo) will be prepared, packaged and labelled by Tayside Pharmaceuticals. The medication will come labelled as “Participant 1”, “Participant 2”, etc. and will be distributed to the participant by the research fellow according to their sequence number. Randomisation will be carried out by Tayside Pharmaceuticals using block randomisation. They will use a validated randomisation program and will securely backup both the randomisation seed and the randomisation allocation. Two copies of the allocation will be supplied with the IMP & placebo tablets to the Clinical Trials Pharmacy, Ninewells, and to the Clinical Trial Pharmacy at Western Infirmary, Glasgow who operate a 24 hour emergency unblinding facility.

### Treatment Allocation

Participants will be given blinded medication. At the randomisation visit they will be dosed with either allopurinol 100mg or matched placebo.

They will then be asked to take one tablet after each dialysis session for two weeks (+/-2 days).

Thereafter the medication will be increased weekly (after 3 dialysis sessions) as tolerated (+/- 2 days) to the dose decided in the pilot study – ie one week at 200mg, one week at 250mg, one week at 300mg and upto 350mg, or placebo as determined by pilot data.

Compliance will be checked and documented using tablet counts at each visit. If non-compliant, they will be encouraged to become compliant. If they persist as non compliant, (<70% compliant) they will stay in the study, but not on study medication, in order to do an “intention to treat” analysis.

### Emergency Unblinding Procedures

A clinician familiar with the research (and named on the delegation log) will be available each workday for contact by all participants. This will include the CI, the PI and a nominated other research fellow based within the Division of Cardiovascular & Diabetes Medicine department at Ninewells and at BHF Cardiovascular Research Centre, University of Glasgow. Any other clinician who sees participants in the study will be free to stop the study drug if they feel it is clinically indicated and may contact the Clinical Trials Pharmacy Department, Ninewells or at the Western Infirmary in Glasgow to break the code if they feel it is necessary. However side effects tend to be minor (see section 6 below) and therefore emergency unblinding is highly unlikely. The Ninewells and Western Infirmary Pharmacy departments run an out of hours service for doctors to contact, if necessary in emergency. We will follow TASC SOP40 giving details of how to unblind, who can do it and where contacts for unblinding can be found.

### Withdrawal procedures

Side effects are likely to be minor and expected and it will therefore be a participant choice as to whether they withdraw or not from a study. Rash is the main side effect and withdrawal will occur if it is marked as persistent. Participants who withdraw will be replaced if possible within the study timeframe. Contact (by telephone) will be maintained by the research fellow/trial manager/research nurse with those who withdraw to ensure resolution of adverse event(s). The reasons for withdrawal will be noted in the participant’s CRF and casenotes. If withdrawal is due to an AE it will be logged as such on the Adverse Event Log.

Participants are free to withdraw from the study at any time. The reasons, if known, will be recorded in the medical casenotes and CRF.

# INVESTIGATIONAL MEDICINAL PRODUCT

## STUDY DRUG

### Study Drug Identification

Allopurinol Ph Eur 100mg, 200mg, 250mg, 300mg and 350mg enclosed in a hard gelatine capsule pack.

Placebo capsules containing Lactose Ph Eur to match the actives in a hard gelatine capsule pack.

### Study Drug Manufacturer

Tayside Pharmaceuticals, Ninewells Hospital, Dundee.

### Marketing Authorisation Holder

Not applicable. IMP dossier to be formulated by Tayside Pharmaceuticals

### Labelling and Packaging

Tayside Pharmaceuticals, Ninewells Hospital, Dundee.

### Storage

Drugs will be stored in the Clinical Trial Pharmacy (Ninewells Hospital) and a small supply in the Division of Cardiovascular and Diabetes Medicine Local Drug Storage Area. This latter area is subject to audit by the Clinical Trials Pharmacy, Ninewells Hospital. Two sets of drug accountability logs will be held. Storage in Glasgow will by via the Clinical Trials Pharmacy at Western Infirmary Glasgow.

### Summary of Product Characteristics

The Summary of Product Characteristics (SmPC) will be filed appropriately in the Trial Master File (TMF), ISFs and Pharmacy Site File (PSF) and be updated regularly. Allopurinol will however be given outwith its usual clinical use (off-label).

### Accountability procedures

These will be overseen by the Clinical Trials Pharmacist in the Clinical Trials Pharmacy Department (Ninewells Hospital). This will be documented as such in the TMF and Accountability Logs will be held in the TMF. We will follow the standard TASC policy on drug accountability in CTIMPs.

## STUDY COMPARATOR

### Comparator Identification

Placebo capsules (with an identical appearance to the active drug) and containing only lactose.

### Comparator Manufacturer

Tayside Pharmaceuticals, Ninewells Hospital, Dundee.

### Labelling and Packaging

Tayside Pharmaceuticals, Ninewells Hospital, Dundee.

### Storage

Drugs will be stored in the Clinical Trial Pharmacy (Ninewells Hospital) and a small supply in the Division of Cardiovascular and Diabetes Medicine Local Drug Storage Area. This latter area is subject to audit by the Clinical Trials Pharmacy, Ninewells Hospital. Two sets of drug accountability logs will be held. Storage in Glasgow will by via the Clinical Trials Pharmacy at Western Infirmary Glasgow.

## DOSING REGIME

Participants will be given blinded medication. At the randomisation visit they will be dosed with either allopurinol 100mg or matched placebo.

They will then be asked to take one tablet after each dialysis session for two weeks (+/-2 days).

Thereafter the medication will be increased weekly as tolerated (+/- 2 days) to the dose decided in the phase 1 dose escalation study – ie one week at 200mg, one week at 250mg, one week at 300mg and upto 350mg, or placebo as determined by pilot data.

Compliance will be checked and documented using tablet counts at each visit. If non compliant, they will be encouraged to become compliant. If they persist as non compliant (<80% compliance), they will stay in the study, but not on study medication, in order to do an “intention to treat” analysis.

## DOSE CHANGES

If there is any significant deterioration in U&Es or LFTs, as considered by a trial physician, then the dose will be stopped and they will not receive any further IMP. If the participant is taken off the study their GP and consultant nephrologist will be informed of the abnormal blood results or further monitoring though the patient will remain in the study for the entire duration even if study medications are stopped.

**.**

## PARTICIPANT COMPLIANCE

Compliance will be checked and documented using tablet counts at each visit. Should compliance be poor the patient will be advised by the research fellow on the importance of correct compliance. Should compliance be less than 70%, they will be withdrawn from treatment.

## OVERDOSE

This is unlikely given the simple once daily dosing but there is no specific antidote. As noted in the SmPC, ingestion of up to 22.5 g allopurinol (i.e., the equivalent of 64 maximum dose 350mg study drug capsules) without adverse effect has been reported. Symptoms and signs including nausea, vomiting, diarrhoea and dizziness have been reported in a patient who ingested 20g allopurinol. Recovery followed general supportive measures. Massive absorption of allopurinol may lead to considerable inhibition of xanthine oxidase activity, which should have no untoward effects unless affecting concomitant medication, especially with 6-mercaptopurine and/or azathioprine. Adequate hydration to maintain optimum diuresis facilitates excretion of allopurinol and its metabolites. If considered necessary further haemodialysis may be used.

## OTHER MEDICATIONS

### Permitted Medications

Other medications (save for those listed in 6.7.2) will be allowed. All concurrent medication(s) will be recorded.

### Prohibited Medications

6-mercaptopurine or azathioprine: concurrent prescription of either of these drugs would not be allowed due to the known interaction of these drugs with allopurinol. Any participants already on these drugs would be excluded at screening. If they needed to start on treatment with either of these whilst receiving study medication then they would be withdrawn from study.

Warfarin: due to the possible influence of allopurinol on warfarin levels (requiring increased frequency of monitoring) any participants already on a stable dose of this drug would be excluded at screening. If they needed to start on treatment with warfarin whilst receiving study medication then this would however still be possible (as any patient newly commenced on warfarin has frequent monitoring of their INR).

Theophylline: due to the possible influence of allopurinol on theophylline levels any participants already on this drug would be excluded at screening. If they needed to start on treatment with theophylline whilst receiving study medication then they would be withdrawn from study.

Ampicillin/amoxicillin: not prohibited, but an increase in frequency of skin rash has been reported among s receiving ampicillin or amoxicillin concurrently with allopurinol compared to patients who are not receiving both drugs. The cause of the reported association has not been established. However, in participants receiving allopurinol an alternative to ampicillin or amoxicillin will be used where available.

The GP information letter and PIS will contain information on all the above points.

### Concurrent Medications

**Details of all con-meds will be recorded in the CRF.**

# STUDY ASSESSMENTS

## STUDY VISITS

(***See Appendix 2 ALTERED Participant study schedule)***

As outlined in section 5.2, participants will be provided with a full PIS and are given at least 24 hours to consider their response before a screening visit will be arranged for consent to be taken and to check their eligibility by all the criteria.

**Phase 1 Dose Escalation Study**

- **Visit 1 –Screening visit 1**
  - Participant consent– answer any outstanding questions and complete consent form.
  - Clinical Examination, Medical and Family history
  - Baseline Urate bloods
  - Safety bloods
  - Record list of current medications
- **Visit 2 Randomisation visit (Day 0) upto 4 week post screening**
  - Dosing with Allopurinol
  - Supply of 1^st^ dose study medication allopurinol 100mg x 3 dialysis sessions 7 days (+/- 1 dialysis session)
  - Record list of current medications
  - Vital signs
  - Assess any adverse events from screening visit to randomisation
  - Safety bloods
- **Visit 3- Progress visit at 4^th^ Dialysis Session**
  - Review urate and safety bloods
  - If urate decreased by >50%- **no further visits**
  - If urate decreased by <50%- titrate up to 200mg allopurinol
  - Stat Dose 200mg Allopurinol
  - Supply of 2nd dose study medication allopurinol 200mg x 3 dialysis sessions (+/- 1 dialysis session)
  - Record list of current medications
  - Vital signs
  - Assess any adverse events from visit 2 to visit 3
  - Check drug compliance
- **Visit 4- Progress visit at 7^th^ Dialysis Session**
  - Review urate and safety bloods
  - If urate decreased by >50% from screening - **no further visits**
  - If urate decreased by <50%- titrate up to 250mg allopurinol
  - Stat Dose 250mg Allopurinol
  - Supply of 3rd dose study medication allopurinol 250mg x 3 dialysis sessions (+/- 1 dialysis session)
  - Record list of current medications
  - Vital signs
  - Assess any adverse events from visit 3 to visit 4
  - Check drug compliance
- **Visit 5- Progress visit at 10^th^ Dialysis Session**
  - Review urate and safety bloods
  - If urate decreased by >50% from screening - **no further visits**
  - If urate decreased by <50%- titrate up to 300mg allopurinol
  - Stat Dose 300mg Allopurinol
  - Supply of 4th dose study medication allopurinol 300mg x 3 dialysis sessions (+/- 1 dialysis session)
  - Record list of current medications
  - Vital signs
  - Assess any adverse events from visit 4 to visit 5
  - Check drug compliance
- **Visit 6- Progress visit at 13^th^ Dialysis Session**
  - Review urate and safety bloods
  - If urate decreased by >50% from screening - **no further visits**
  - If urate decreased by <50%- titrate up to 350mg allopurinol
  - Stat Dose 350mg Allopurinol
  - Supply of 5th dose study medication allopurinol 300mg x 3 dialysis sessions (+/- 1 dialysis session)
  - Record list of current medications
  - Vital signs
  - Assess any adverse events from visit 5 to visit 6
  - Check drug compliance
- **Visit 7- Final visit at 16^th^ Dialysis Session (+/- 1 dialysis session)**
  - Review urate and safety bloods
  - Record list of current medications
  - Vital signs
  - Assess any adverse events from visit 4 to visit 5
  - Check drug compliance

**Phase 2 ALTERED Trial**

- **Visit 1 – Screening visit 1**
  - Participant consent– answer any outstanding questions and complete consent form.
  - Clinical Examination, Medical and Family history
  - Baseline Urate bloods
  - ECG
  - Safety bloods
  - Echocardiogram if not done in previous 2 years
  - MRI Scan –(***Only if Echo demonstrates LVH)***
  - 24hr BP monitoring
  - Record list of current medications
- **Visit 2 – Baseline visit (Day 0) upto 4 week post screening-**
  - Vital signs
  - Dosing with Allopurinol 100mg/placebo
  - Supply of 1^st^ dose study medication allopurinol 100mg x 6 dialysis sessions (+/- 1 dialysis session)
  - Record list of current medications
  - Assess any adverse events from screening visit to randomisation
  - Safety & research bloods
  - FMD & PWV measurements if participant agrees

**N*ote visits 3a- 3d are dependent on pilot data deciding eventual optimum dose of allopurinol***

**Visit 3a- Progress visit (Week 2 +/- 1 dialysis session)**

- - Supply of 2nd dose study medication allopurinol 200mg/placebo x 3 dialysis sessions (+/- 1 dialysis session)
  - Record list of current medications
  - Vital signs
  - Assess any adverse events from visit 2 to visit 3a
  - Check drug compliance
  - Safety blood tests
- **Visit 3b Progress visit (Week 3 +/- 1 dialysis session)**
  - Supply of 3^rd^ dose study medication allopurinol 250mg/placebo x 3 dialysis sessions (+/- 1 dialysis session)
  - Record list of current medications
  - Vital signs
  - Safety bloods
  - Assess any adverse events from visit 3a to visit 3b
  - Check drug compliance
- **Visit 3c-Progress visit (Week 4 +/- 1 dialysis session)**
  - Supply of 4th dose study medication allopurinol 300mg/placebo x 3 dialysis sessions (+/- 1 dialysis session)
  - Record list of current medications
  - Vital signs
  - Safety bloods
  - Assess any adverse events from visit 3b to visit 3c
  - Check drug compliance
- **Visit 3d-Progress visit (Week 5 +/- 1 dialysis session)**
  - Supply of 5th dose study medication allopurinol 350mg/placebo x 3 dialysis sessions (+/- 1 dialysis session)
  - Record list of current medications
  - Vital signs
  - Safety bloods
  - Assess any adverse events from visit 3c to visit 3d
  - Check drug compliance
- **Visit 4- Progress visit (week 6 +/- 1 dialysis session)**
  - Dispense study medication allopurinol at optimum dose/placebo x 5 months
  - Record list of current medications
  - Vital signs
  - Safety bloods
  - Assess any adverse events from visit 3 to visit 4
  - Check drug compliance
- **Visit 5- Progress visit (Month 6 +/- 2 weeks)**
  - Dispense study medication allopurinol/placebo x 3 months
  - Record list of current medications
  - Vital signs
  - Safety bloods
  - Assess any adverse events from visit 4 to visit 5
  - Check drug compliance
- **Visit 6- Progress visit (Month 9 +/- 2 weeks)**
  - Dispense study medication allopurinol/placebo x 3 months
  - Record list of current medications
  - Safety bloods
  - Vital signs
  - Assess any adverse events from visit 5 to visit 6
  - Check drug compliance
  - FMD measurements
  - PWV measurements
- **Visit 7- Final visit (Month 12 +/- 2 weeks)**
  - Review urate and safety bloods
  - Record list of current medications
  - Safety bloods
  - Vital signs
  - Assess any adverse events from visit 5 to visit 6
  - Check drug compliance
  - FMD measurements
  - PWV measurements
  - ECG
  - MRI Scan – may be done upto 2 week post visit

## SAFETY ASSESSMENTS

**See Section 7.1 above**

# DATA COLLECTION & MANAGEMENT

## Data Collection

The data will be collected by the research fellow or trial manager on paper with subsequent transcription to electronic form. Electronic storage will be in an encrypted form on a password protected device.

The medical notes will act as source data for past medical history & blood results. ECGs are printed and filed in medical notes.

## Data Management System

A data management system will be provided by TCTU using OpenClinica.  The study system will be based on the protocol and CRF for the study and individual requirements of the investigators.  Development and validation of the study database; and QC and extraction of data will be done according to TCTU procedures.  Extracts for analysis will be based on the dummy data tables provided by the study team.

# STATISTICS AND DATA ANALYSIS

## SAMPLE SIZE CALCULATION

**POWER CALCULATIONS**

In powering MRI studies of LVH regression, it is recommended that they are powered for a 10gm change in LV mass (= 4.8g/m^2^ change in LVMI). Grothues et al [[34](#_ENREF_34)] recommend this as this size of effect should reduce future CV events. We have powered this study based on two published studies where 12 months of treatment were given, as planned here. In Edwards et al [[35](#_ENREF_35)], there was a 10g reduction in LV mass with a standard deviation of 12g in CKD patients. Based on these values, we would need 32 patients per group to have 90% power at p<0.05 to detect this magnitude of change in LV mass. In the only MRI study which we have done involving one year of treatment, we saw an LV mass fall of 14 14 g, which would give us a very similar power calculation.[[36](#_ENREF_36)] (Unfortunately our allopurinol in mild CKD study only involved 9 months treatment and time is known to be crucial when a treatment is trying to regress LV mass : a larger effect is anticipated here with 12 months therapy). If we anticipate a 10% death or transplantation rate/year and a 10% dropout rate, then we will need a total of 76 patients to be recruited. Glasgow has 650 dialysis patients and Dundee 200, so that our planned recruitment should be achievable, especially since these patients are well aware of their poor prognosis and hence usually very willing to volunteer for potentially lifesaving research from which they may well personally benefit in the long term.


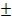

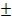


## PROPOSED ANALYSES

**Statistical Analysis**

Statistical analysis will be performed using SPSS (SPSS, Chicago, USA). Data will be expressed as mean ± SD unless stated otherwise. One-way ANOVA or χ^2^ test will be used to determine the significance of differences between both groups (normally distributed variable). Analysis of covariance will also be performed using the month 12 value as the dependent variable and the baseline value treated as a covariate, along with age, SBP, and DBP, to account for any differences in baseline measures of the primary and secondary outcomes. A *P* value <0.05 will be considered significant.

A detailed statistical analysis plan will be prepared during the trial.

## MISSING DATA

**This is an intention to treat study so all non compliers, withdrawn patients or missing data will be analysed by imputation. We will also do a completed cases only analysis.**

# ADVERSE EVENTS

Adverse event reporting will be carried out in accordance with TASC SOP 11: Identifying, Recording and Reporting Adverse Events for Clinical Trials of Investigational medicinal Products. The CI (or delegate) is responsible for the detection and documentation (and reporting, where required) of adverse events

The IMP (investigational medicinal product) reference document to be used when assessing causality will be the SmPC.

All reported adverse events (AEs) will be recorded in detail on an adverse event CRF page For all AEs, the Investigator will initiate the appropriate treatment according to their medical judgment.

## DEFINITIONS

An **adverse event** (AE) is any untoward medical event affecting a clinical trial participant. Each initial AE will be considered for severity, causality or expectedness and may be reclassified as a serious event or reaction based on prevailing circumstances.

An **adverse reaction** (AR) is where it is suspected that an AE has been caused by a reaction to a trial drug

A **serious adverse event** (SAE), **serious adverse reaction** (SAR) or **suspected unexpected serious adverse reaction** (SUSAR) is any AE, AR or UAR that at any dose:

- results in death
- is life threatening
- requires hospitalisation or prolongation of existing hospitalisation
- results in persistent or significant disability or incapacity
- is a congenital anomaly or birth defect
- Or is otherwise considered serious

**Note:** Hospitalisations for treatment planned prior to randomisation and hospitalisation for elective treatment of a pre-existing condition **will not** be considered as an SAE. Additionally hospitalisations related to shunt problems, infections, and cardiovascular events such as heart failure or acute coronary syndrome will not be considered as SAEs though will be noted as adverse events and recorded. Any adverse events occurring during such hospitalisation must though be recorded.

## DETECTING AEs AND SAEs

The CI or PI are responsible for the detection and documentation of events meeting the criteria and definitions detailed below.

Full details of contraindications and side effects that have been reported following administration of the trial drug can be found in the SmPC. Adverse reactions in association with allopurinol are rare in the overall treated population and mostly of a minor nature. The incidence is higher in the presence of renal and/or hepatic disorder (hence why UEs and LFTs will be monitored).

Participants should be instructed to contact their Investigator at any time after consenting to join the trial if any symptoms develop. All reported adverse events (AEs) that occur after joining the trial will be recorded in detail in the CRF AE log. In the case of an AE, the Investigator should initiate the appropriate treatment according to their medical judgment. Participants with AEs present at the last visit must be followed up until resolution of the event.

It is worth noting also that these patients attend dialysis sessions three times per week and are assessed by trained nursing and medical staff at each visit who will know of their participation in this clinical trial. These staff will have direct access to the PI or CI and will make them aware of any medical conditions of concern that can be assessed by the PI or CI for relationship to the study medications.

## RECORDING AEs AND SAEs

Depending on severity, when an AE occurs, the CI or delegate will review all documentation (e.g. hospital notes, laboratory and diagnostic reports) related to the event. The CI or delegate will then ensure that all relevant information is recorded on the adverse event CRF page. If the event is an SAE, SAR or SUSAR, an SAE form must also be completed and sent to the Sponsor Pharmaco-vigilance Section.

Information to be collected will include dose, type of event, onset date, Investigator assessment of severity and causality, date of resolution as well as treatment required, investigations needed and outcome.

## EVALUATION OF AEs AND SAEs

Seriousness, causality, severity and expectedness will be evaluated as though the participant is taking an active drug.

### Assessment of Seriousness

The CI or delegate should make an assessment of seriousness as defined in Section 10.1.

### Assessment of Causality

The CI or delegate will make an assessment of whether the AE/SAE is likely to be related to treatment according to the following definitions:

**Unrelated**: where the AE is not considered to be related to the study drug.

**Possibly**: although a relationship to the study drug cannot be completely ruled out, the nature of the event, the underlying disease, concomitant medication or temporal relationship make other explanations more likely.

**Probably**: the temporal relationship and absence of a more likely explanation suggest the event could be related to the study drug.

**Definitely**: the known effects of the study drug or its therapeutic class, or based on challenge testing, suggest that the study drug is the most likely cause.

All AEs/SAEs judged as having a reasonable suspected causal relationship (e.g. possibly, probably, definitely) to the study drug will be considered as ARs/SARs. All AEs/SAEs judged as being related (e.g. possibly, probably, definitely) to an interaction between the study drug and another drug will also be considered to be ARs/SAR.

Alternative causes such as natural history of the underlying disease, concomitant therapy, other risk factors and the temporal relationship of the event to the treatment will be considered. The blind will not be broken for the purpose of making this assessment.

### Assessment of Severity

The CI or delegate will make an assessment of severity for each AE/SAE according to one of the following categories:

**Mild**: an event that is easily tolerated by the participant, causing minimal discomfort and not interfering with every day activities.

**Moderate**: an event that is sufficiently discomforting to interfere with normal everyday activities.

**Severe**: an event that prevents normal everyday activities.

Note: the term ‘severe’, used to describe the intensity, should not be confused with ‘serious’ which is a regulatory definition based on participant/event outcome or action criteria. For example, a headache may be severe but not serious, while a minor stroke is serious but may not be severe.

### Assessment of Expectedness

If an event is judged to be an AR/SAR, the evaluation of expectedness will be made based on knowledge of the reaction and the relevant product information documented in the SmPC.

ARs/SARs may be classed as either:

**Expected:** the AR is consistent with the toxicity of the study drug listed in the SmPC.

**Unexpected:** the AR is not consistent with the toxicity in the SmPC.

## REPORTING OF SAEs/SARs/SUSARs

SAEs must be reported to the TASC Pharmacovigilance section within 24 hours of the CI or delegate becoming aware of the event. The TASC SAE form must be completed as thoroughly as possible with all available details of the event and signed by the Investigator. If any of the information is not available at the time of reporting, the Investigator will ensure that any missing information is provided on a follow up SAE form as soon as this becomes available. It will be indicated on the form that this information is follow-up information of a previously reported event.

All SAEs must be followed up to resolution and any additional information reported to the TASC Pharmacovigilance Section using the TASC SAE Form.

## REGULATORY REPORTING REQUIREMENTS

Development Safety Update Reporting (DSUR) will be carried out in accordance with the relevant TASC SOP: development update safety reporting in Clinical trials of Investigational Medicinal Products. An annual safety report must be submitted to the MHRA and REC on the anniversary of the clinical trial authorisation and annually thereafter. The development update safety report (DUSR) will be prepared by the TASC Pharmacovigilance section. The CI or delegate will approve and sign the (DUSR), which will then be submitted to the MHRA and the REC by the TASC Pharmacovigilance Section.

The Sponsor is responsible for reporting SUSARs to the MHRA and to the REC. Fatal or life threatening SUSARs must be reported within 7 days and other non fatal SUSARs within 15 days. SUSARs should be reported to the MHRA using the electronic SUSAR reporting tool. SUSARs should be reporting to the REC using the NRES Safety Report Form (CTIMPs) and enclosing the SUSAR report.

## URGENT SAFETY MEASURES

The following safety issues should also be reported by the Investigator to the Sponsor in an expedited fashion:

- An increase in the rate of occurrence or a qualitative change of expected SAR, which is judged to be clinically important.
- Post-study SUSARs that occur after the trial participant has completed a clinical trial and are notified to the Investigator.
- New events related to the trial or the development of the investigational medicinal products and are likely to affect the safety of the participants.
- Recommendations of the Data Monitoring Committee where relevant for the safety of trial participants.

The Sponsor is responsible for informing the MHRA and the REC of these safety issues.

# PREGNANCY

Pregnancy is not considered an AE or SAE, however an abnormal outcome would be. If a pregnancy occurs, the CI or delegate must collect pregnancy information for female trial participants or female partners of male trial participants who become pregnant while participating in a study.

For female partners of male trial participants who become pregnant while participating in a study, additional consent should be obtained to follow up the pregnancy.

The CI or delegate should complete the TASC Pregnancy Notification Form and submit to the TASC Pharmacovigilance section within 14 days of being made aware of the pregnancy.

Any pregnancy should be followed to outcome. Pregnancy follow up information must be recorded on the TASC Pregnancy Follow up form and submitted to the TASC Pharmacovigilance section.

# TRIAL MANAGEMENT AND OVERSIGHT ARRANGEMENTS

## TRIAL MANAGEMENT GROUP

The trial will be coordinated by a Project Management Group, consisting of the grantholders, including the CI, and the research fellow. (See Appendix 1)

## TRIAL MANAGEMENT

The research fellow will oversee the study on a day-to-day basis and will be accountable to the CI. The CI is responsible for the overall conduct of the study and will be responsible for checking the CRFs for completeness, accuracy and consistency. Any queries will be resolved by a delegated member of the trial team.

A Delegation Log will be prepared, detailing the responsibilities of each member of staff working on the trial.

## TRIAL STEERING COMMITTEE

The Project Management Group will also act as the Trial Steering Committee (TSC).

## DATA MONITORING COMMITTEE

This is not felt necessary as this is a relatively small study involving an already marketed drug. The research group have also previously done many similar studies with allopurinol with no major problems.

## INSPECTION OF RECORDS

The CI, PIs and all institutions involved in the study will permit trial related monitoring, audits, REC review, and regulatory inspection(s). In the event of an audit, the CI agrees to allow the Sponsor, representatives of the Sponsor or regulatory authorities direct access to all study records and source documentation.

## RISK ASSESSMENT

A pre-Sponsorship study risk assessment will be carried out by the TASC Research Governance Manager prior to Sponsorship approval being granted.

## STUDY MONITORING

Study monitoring will be accordance with TASC SOP 03.

The purposes of trial monitoring are to verify that:

a) The rights and well-being of human subjects are protected.

b) The reported trial data are accurate, complete, and verifiable from source documents.

c) The conduct of the trial is in compliance with the currently approved protocol/amendment(s), with GCP, and with the applicable regulatory requirement(s).

The sponsor will determine the appropriate extent and nature of monitoring and will appoint appropriately qualified and trained monitors.

The monitor will communicate any monitoring findings to both the CI (or PI if multicentre study) and the sponsor.

### Potential Risks

Potential adverse reaction to allopurinol as outlined in the SmPC and discussed in section 6.

Precipitation of attack of gout upon commencement of treatment.

### Minimising Risk

Careful monitoring of patient’s blood results throughout the trial and dedicated care and adverse event monitoring will be undertaken as required.

# GOOD CLINICAL PRACTICE

## ETHICAL CONDUCT OF THE STUDY

The study will be conducted in accordance with the principles of Good Clinical Practice (GCP).

After Sponsorship approval is obtained, a favorable ethical opinion will be obtained from the appropriate REC and appropriate NHS R&D approval(s) will be obtained prior to commencement of the study.

## REGULATORY COMPLIANCE OF THE STUDY

Regulatory approval will be obtained from the appropriate Regulatory Authority. The protocol and study conduct will comply with the Medicines for Human Use (Clinical Trials) Regulations 2004, and any relevant amendments.

## CI RESPONSIBILITIES

The CI will be responsible for the overall conduct of the study at the site and compliance with the protocol and any protocol amendments. In accordance with the principles of GCP, the following areas listed in this section are also the responsibility of the CI. Responsibilities may be delegated to an appropriate member of study staff at each site. Delegated tasks must be documented on a Delegation Log and signed by all those named on the list and by the CI or, for multi-site studies, the local PI.

### Informed Consent

The CI will be responsible for ensuring informed consent is obtained before any protocol specific procedures are carried out as the decision of a participant to participate in clinical research is voluntary and should be based on a clear understanding of what is involved.

Participants will receive adequate oral and written information – appropriate Participant Information Sheet (PIS) and Informed Consent Form (ICF) will be provided. The oral explanation to the participant will be performed by the CI or designate and will cover all the elements specified in the PIS and ICF.

The participant will be given every opportunity to clarify any points they do not understand and, if necessary, ask for more information. The participant will be given sufficient time to consider the information provided. It will be emphasised that the participant may withdraw their consent to participate at any time without loss of benefits to which they otherwise would be entitled.

The participant will be informed and must agree to their medical records being inspected by regulatory authorities, REC and monitors but it will be explained that their name will not be disclosed outside the study site.

The CI or delegate and the participant will sign and date the ICF to confirm that consent has been obtained. The participant will receive a copy of this document and a copy will be filed in their medical case note, where appropriate, TMF or CRF and Investigator Site File (ISF) or CRF as appropriate.

### Study Staff

The CI and other appropriate study staff will be familiar with the IMP, protocol and the study requirements. The CI will ensure that all staff assisting with the study are adequately informed and trained about the IMP, protocol and their trial related duties as appropriate.

### Data Recording

The CI is responsible for the quality of the data recorded in the CRF.

### CI Documentation

Prior to beginning the study, the CI will be asked to provide particular essential documents to the Sponsor, including but not limited to:

- An original signed Investigator’s Declaration (as part of the Clinical Trial Agreement documents);
- Curriculum vitae (CV) signed and dated by the CI indicating that it is accurate and current.

The CI, with the agreement of the Sponsor, will ensure all other documents required for compliance with the principles of GCP are retained in the TMF and that appropriate documentation is available in site ISFs.

### GCP Training

All study staff will hold evidence of appropriate GCP training or undergo GCP training. This will be updated around every two years throughout the trial.

### Confidentiality

All laboratory specimens, evaluation forms, reports, and other records will be identified in a manner designed to maintain participant confidentiality. All records will be kept in a secure storage area with limited access to study staff only. Clinical information will not be released without the written permission of the participant, except as necessary for monitoring and auditing by the Sponsor, its designee or Regulatory Authorities. The CI and study staff involved with this study will not disclose or use for any purpose other than performance of the study, any data, record, or other unpublished, confidential information disclosed to those individuals for the purpose of the study. Prior written agreement from the Sponsor or its designee will be obtained for the disclosure of any said confidential information to other parties.

### Data Protection

The CI and study staff involved with this study will comply with the requirements of the Data Protection Act 1998 with regard to the collection, storage, processing and disclosure of personal information and will uphold the Act’s core principles. Access to collated participant data will be restricted to those clinicians treating the participants.

Computers used to collate the data will have limited access measures via user names and passwords.

Published results will not contain any personal data that could allow identification of individual participants.

# STUDY CONDUCT RESPONSIBILITIES

## PROTOCOL AMENDMENTS

Any changes in study activity, except those necessary to remove an apparent, immediate hazard to the participant, will be reviewed and approved by the CI. Amendments to the protocol will be submitted in writing to the Sponsor for approval prior to being submitted to the appropriate REC, Regulatory Authority and local NHS R&D for approval. All approvals will be in place prior to participants being enrolled into an amended protocol.

## PROTOCOL VIOLATIONS AND DEVIATIONS

The CI will not implement any deviation from the protocol without agreement from the Sponsor, appropriate REC, Regulatory Authority and without local NHS R&D approval, except where necessary to eliminate an immediate hazard to trial participants.

In the event that any study Investigator needs to deviate from the protocol, the nature of and reasons for the deviation will be recorded in the CRF, documented in a TASC Breaches & Deviations Log and submitted to the Sponsor. If this necessitates a subsequent protocol amendment, this will be submitted to the Sponsor for approval and then to the appropriate REC, Regulatory Authority and local NHS R&D for review and approval as appropriate.

## STUDY RECORD RETENTION

TASC SOP 13 will be used for the study. As the data from this study does not form part of an application for a Marketing Authorisation (MA) all study documentation, including medical case notes, will be kept for at least 5 years.

## END OF STUDY

The end of study is defined as the last participant last visit (LPLV)

The CI and/or the TSC have the right at any time to terminate the study for clinical or administrative reasons.

The end of the study will be reported to the Sponsor, REC and Regulatory Authority within 90 days, or 15 days if the study is terminated prematurely. The CI will ensure that any appropriate follow up is arranged for all participants.

A summary report of the study will be provided to the Sponsor, REC and Regulatory Authority within 1 year of the end of the study.

## CONTINUATION OF DRUG FOLLOWING THE END OF STUDY

This is not appropriate since confirmatory studies are necessary before guidelines for this condition would recommend this particular therapy.

# REPORTING, PUBLICATIONS AND NOTIFICATION OF RESULTS

## AUTHORSHIP POLICY

Ownership of the data arising from this study resides with the study team and their respective employers. On completion of the study, the study data will be analysed and tabulated, and a clinical study report will be prepared.

## PUBLICATION

The clinical study report will be used for publication and presentation at scientific meetings. Investigators have the right to publish orally or in writing the results of the study.

Summaries of results will also be made available to Investigators for dissemination within their clinics (where appropriate and according to their discretion).

## PEER REVIEW

Peer review of the protocol will occur via the Sponsorship Committee and of the resulting publication by the referees of the journal to which the paper (and its protocol) will be submitted.

This study has been funded by the British Heart Foundation who have peer reviewed the grant application.

# REFERENCES

1. Foley RN, Parfrey PS, Sarnak MJ. Clinical epidemiology of cardiovascular disease in chronic renal disease. Am J Kidney Dis. 1998 Nov;32(5 Suppl 3):S112-9.

2. Foley RN, Parfrey PS, Harnett JD, Kent GM, Martin CJ, Murray DC, et al. Clinical and echocardiographic disease in patients starting end-stage renal disease therapy. Kidney Int. 1995 Jan;47(1):186-92.

3. Paoletti E, Bellino D, Cassottana P, Rolla D, Cannella G. Left ventricular hypertrophy in nondiabetic predialysis C KD. Am J Kidney Dis. 2005 Aug;46(2):320-7.

4. Takimoto E, Kass DA. Role of oxidative stress in cardiac hypertrophy and remodeling. Hypertension. 2007 Feb;49(2):241-8.

5. Terawaki H, Yoshimura K, Hasegawa T, Matsuyama Y, Negawa T, Yamada K, et al. Oxidative stress is enhanced in correlation with renal dysfunction: examination with the redox state of albumin. Kidney Int. 2004 Nov;66(5):1988-93.

6. Boaz M, Smetana S, Weinstein T, Matas Z, Gafter U, Iaina A, et al. Secondary prevention with antioxidants of cardiovascular disease in endstage renal disease (SPACE): randomised placebo-controlled trial. Lancet. 2000 Oct 7;356(9237):1213-8.

7. Levy D, Garrison RJ, Savage DD, Kannel WB, Castelli WP. Prognostic implications of echocardiographically determined left ventricular mass in the Framingham Heart Study. N Engl J Med. 1990 May 31;322(22):1561-6.

8. Liao Y, Cooper RS, McGee DL, Mensah GA, Ghali JK. The relative effects of left ventricular hypertrophy, coronary artery disease, and ventricular dysfunction on survival among black adults. JAMA. 1995 May 24-31;273(20):1592-7.

9. Stack AG, Saran R. Clinical correlates and mortality impact of left ventricular hypertrophy among new ESRD patients in the United States. Am J Kidney Dis. 2002 Dec;40(6):1202-10.

10. Haider AW, Larson MG, Benjamin EJ, Levy D. Increased left ventricular mass and hypertrophy are associated with increased risk for sudden death. J Am Coll Cardiol. 1998 Nov;32(5):1454-9.

11. Houghton JL, Frank MJ, Carr AA, von Dohlen TW, Prisant LM. Relations among impaired coronary flow reserve, left ventricular hypertrophy and thallium perfusion defects in hypertensive patients without obstructive coronary artery disease. J Am Coll Cardiol. 1990 Jan;15(1):43-51.

12. Nakashima Y, Nii T, Ikeda M, Arakawa K. Role of left ventricular regional nonuniformity in hypertensive diastolic dysfunction. J Am Coll Cardiol. 1993 Sep;22(3):790-5.

13. Verdecchia P, Reboldi G, Gattobigio R, Bentivoglio M, Borgioni C, Angeli F, et al. Atrial fibrillation in hypertension: predictors and outcome. Hypertension. 2003 Feb;41(2):218-23.

14. Levy D, Salomon M, D'Agostino RB, Belanger AJ, Kannel WB. Prognostic implications of baseline electrocardiographic features and their serial changes in subjects with left ventricular hypertrophy. Circulation. 1994 Oct;90(4):1786-93.

15. Wachtell K, Okin PM, Olsen MH, Dahlof B, Devereux RB, Ibsen H, et al. Regression of electrocardiographic left ventricular hypertrophy during antihypertensive therapy and reduction in sudden cardiac death: the LIFE Study. Circulation. 2007 Aug 14;116(7):700-5.

16. Okin PM, Wachtell K, Devereux RB, Harris KE, Jern S, Kjeldsen SE, et al. Regression of electrocardiographic left ventricular hypertrophy and decreased incidence of new-onset atrial fibrillation in patients with hypertension. Jama. 2006 Sep 13;296(10):1242-8.

17. Okin PM, Devereux RB, Harris KE, Jern S, Kjeldsen SE, Julius S, et al. Regression of electrocardiographic left ventricular hypertrophy is associated with less hospitalization for heart failure in hypertensive patients. Ann Intern Med. 2007 Sep 4;147(5):311-9.

18. Mancini GB, Dahlof B, Diez J. Surrogate markers for cardiovascular disease: structural markers. Circulation. 2004 Jun 29;109(25 Suppl 1):IV22-30.

19. Schillaci G, Pirro M, Mannarino E. Left ventricular hypertrophy reversal and prevention of diabetes: two birds with one stone? Hypertension. 2007 Nov;50(5):851-3.

20. London GM, Pannier B, Guerin AP, Blacher J, Marchais SJ, Darne B, et al. Alterations of left ventricular hypertrophy in and survival of patients receiving hemodialysis: follow-up of an interventional study. J Am Soc Nephrol. 2001 Dec;12(12):2759-67.

21. Kao MP, Ang DS, Gandy SJ, Nadir MA, Houston JG, Lang CC, et al. Allopurinol benefits left ventricular mass and endothelial dysfunction in chronic kidney disease. J Am Soc Nephrol. 2011 Jul;22(7):1382-9.

22. Goicoechea M, de Vinuesa SG, Verdalles U, Ruiz-Caro C, Ampuero J, Rincon A, et al. Effect of allopurinol in chronic kidney disease progression and cardiovascular risk. Clin J Am Soc Nephrol. 2010 Aug;5(8):1388-93.

23. Xu X, Hu X, Lu Z, Zhang P, Zhao L, Wessale JL, et al. Xanthine oxidase inhibition with febuxostat attenuates systolic overload-induced left ventricular hypertrophy and dysfunction in mice. J Card Fail. 2008 Nov;14(9):746-53.

24. Laakso JT, Teravainen TL, Martelin E, Vaskonen T, Lapatto R. Renal xanthine oxidoreductase activity during development of hypertension in spontaneously hypertensive rats. J Hypertens. 2004 Jul;22(7):1333-40.

25. Rajendra NS, Ireland S, George J, Belch JJ, Lang CC, Struthers AD. Mechanistic insights into the therapeutic use of high-dose allopurinol in angina pectoris. J Am Coll Cardiol. 2011 Aug 16;58(8):820-8.

26. Baigent C, Landray MJ, Reith C, Emberson J, Wheeler DC, Tomson C, et al. The effects of lowering LDL cholesterol with simvastatin plus ezetimibe in patients with chronic kidney disease (Study of Heart and Renal Protection): a randomised placebo-controlled trial. Lancet. 2011 Jun 25;377(9784):2181-92.

27. Fellstrom BC, Jardine AG, Schmieder RE, Holdaas H, Bannister K, Beutler J, et al. Rosuvastatin and cardiovascular events in patients undergoing hemodialysis. N Engl J Med. 2009 Apr 2;360(14):1395-407.

28. Dalbeth N, Stamp L. Allopurinol dosing in renal impairment: walking the tightrope between adequate urate lowering and adverse events. Semin Dial. 2007 Sep-Oct;20(5):391-5.

29. Patel RK, Mark PB, Macnaught G, Stevens KK, McQuarrie EP, Steedman T, et al. Altered relative concentrations of high-energy phosphates in patients with uraemic cardiomyopathy measured by magnetic resonance spectroscopy. Nephrol Dial Transplant. 2012 Jan 11.

30. Mark PB, Doyle A, Blyth KG, Patel RK, Weir RA, Steedman T, et al. Vascular function assessed with cardiovascular magnetic resonance predicts survival in patients with advanced chronic kidney disease. J Cardiovasc Magn Reson. 2008;10:39.

31. Hirsch GA, Bottomley PA, Gerstenblith G, Weiss RG. Allopurinol Acutely Increases Adenosine Triphospate Energy Delivery in Failing Human Hearts. J Am Coll Cardiol. 2012 February 28, 2012;59(9):802-8.

32. Khatib SY, Farah H, El-Migdadi F. Allopurinol enhances adenine nucleotide levels and improves myocardial function in isolated hypoxic rat heart. Biochemistry (Mosc). 2001 Mar;66(3):328-33.

33. Corretti MC, Anderson TJ, Benjamin EJ, Celermajer D, Charbonneau F, Creager MA, et al. Guidelines for the ultrasound assessment of endothelial-dependent flow-mediated vasodilation of the brachial artery: a report of the International Brachial Artery Reactivity Task Force. J Am Coll Cardiol. 2002 Jan 16;39(2):257-65.

34. Grothues F, Smith GC, Moon JC, Bellenger NG, Collins P, Klein HU, et al. Comparison of interstudy reproducibility of cardiovascular magnetic resonance with two-dimensional echocardiography in normal subjects and in patients with heart failure or left ventricular hypertrophy. Am J Cardiol. 2002 Jul 1;90(1):29-34.

35. Edwards NC, Steeds RP, Stewart PM, Ferro CJ, Townend JN. Effect of spironolactone on left ventricular mass and aortic stiffness in early-stage chronic kidney disease: a randomized controlled trial. J Am Coll Cardiol. 2009 Aug 4;54(6):505-12.

36. Simpson HJ, Gandy SJ, Houston JG, Rajendra NS, Davies JI, Struthers AD. Left ventricular hypertrophy: reduction of blood pressure already in the normal range further regresses left ventricular mass. Heart. 2010 Jan;96(2):148-52.

#### APPENDIX 1: Trial Steering Committee

**Professor Allan Struthers (CI)**

**Dr Elaine Rutherford (PI)**

**Dr Patrick Mark (Study collaborator, NHS Greater Glasgow & Clyde)**

**Sheila Ireland (Trial Manager/Research Nurse)**

**Steve McSwiggan Senior Trial Manager, Tayside Clinical Trials Unit**

#### APPENDIX 2 PHASE 1 Dose ESCALATION study (OPEN LABEL)

**PARTICIPANT STUDY SCHEDULE**

| CLINIC VISIT | **Visit**  **1**  **Screening** | **Visit 2**  **Baseline**  **Dialysis** | **Visit 3**  **(+/- 1 dialysis session)** | **Visit 4**  **(+/- 1 dialysis session)** | **Visit 5**  **(+/- 1 dialysis session)** | **Visit 6**  **(+/- 1 dialysis session)** | **Visit 7**  **Final Visit**  **(+/- 1 dialysis session)** |
| --- | --- | --- | --- | --- | --- | --- | --- |
|  | -1 to -4 weeks | **Day 0** | **4^th^ dialysis Session** | **7^th^ Dialysis session** | **10^th^ Dialysis Session** | **13^th^ Dialysis** | **16^th^ dialysis session** |
| Drug dose |  | 100mg | 200mg | 250mg | 300mg | 350mg | **Stop!** |
| Informed Consent | X |  |  |  |  |  |  |
| Check Inc/Exc Criteria | X |  |  |  |  |  |  |
| Medical History | X |  |  |  |  |  |  |
| Family History | X |  |  |  |  |  |  |
| Clinical Examination | X |  |  |  |  |  |  |
| Demographics | X |  |  |  |  |  |  |
| Vital Signs | X | X | X | X | X | X | X |
| **Urate** | X |  | X | X | X | X | X |
| Safety Blood Tests^A^ | X | X | X | X | X | X | X |
| Dispense Study medication |  | X | X | X | X | X |  |
| Adverse Event Assessment |  | X | X | X | X | X | X |
| Record or Review Con Meds | X | X | X | X | X | X | X |
| Check Drug Compliance |  |  | X | X | X | X | X |

**A: Safety blood tests**: U&Es, LFT’s, FBC, HbA1C, glucose, Calcium, Phosphate

Note that Urate result must be reviewed prior to dispensing of any medication. If urate has decreased by >50% since screening then participant is finished in trial and does not need to continue with higher dose medication.

#### Appendix 3: Main PHASE 2 Randomised Controlled Trial

**Study Schedule ALTERED study**

| CLINIC VISIT | **Visit 1**  **Screening** | **Visit 2**  **Baseline Dialysis** | **Visit 3a** | **Visit 3b** | **Visit 3c** | **Visit 3d** | **Visit 4** | **Visit 5** | **Visit 6** | **Visit 7**  **Final Visit** |
| --- | --- | --- | --- | --- | --- | --- | --- | --- | --- | --- |
|  | **-1 to -4 weeks** | **Day 0** | **Week 2** | **Week 3** | **Week 4** | **Week 5** | **Week 6** | **Month 6** | **Month 9** | **Month 12** |
| Drug dose if increasing |  | **100mg/ placebo** | **200mg/placebo** | **250mg/**  **placebo** | **300mg/**  **placebo** | **350mg/**  **placebo/** |  |  |  |  |
| Informed Consent | X |  |  |  |  |  |  |  |  |  |
| Check Inc/Exc Criteria | X |  |  |  |  |  |  |  |  |  |
| Medical History | X |  |  |  |  |  |  |  |  |  |
| Family History | X |  |  |  |  |  |  |  |  |  |
| Clinical Examination | X |  |  |  |  |  |  |  |  |  |
| Demographics | X |  |  |  |  |  |  |  |  |  |
| Vital Signs | X | X | X | X | X | X | X | X | X | X |
| Echo (if not done in previous 2 years) | X |  |  |  |  |  |  |  |  |  |
| ECG | X |  |  |  |  |  |  |  |  | X |
| MRI# | X |  |  |  |  |  |  |  |  | X |
| Research Bloods, BNP & Urate |  | X |  |  |  |  | X | X | X | X |
| 24hr BP monitoring# | X |  |  |  |  |  |  |  |  | X |
| Safety Blood Tests | X | X | X | X | X | X | X | X | X | X |
| Dispense Study medication |  | X | X | X | X | X | X | X | X |  |
| FMD |  | X |  |  |  |  |  |  | X | X |
| PWV/PWA |  | X |  |  |  |  |  |  | X | X |
| Adverse Event Assessment |  | X | X | X | X | X | X | X | X | X |
| Record or Review Con Meds | X | X | X | X | X | X | X | X | X | X |
| Check Drug Compliance |  |  | X | X | X | X | X | X | X | X |

***A: Safety blood tests:*** *U*&Es, LFT’s, FBC, HbA1C, glucose, calcium, Phosphate**. Note lipids also at Baseline and final visit only.**

***If pilot data identifies a dose of allopurinol to use that is less than 200mg, 250mg, 300mg or 350mg then all steps for subsequent higher doses are removed from study schedule and enough medications are issued upto week 6 visit. #:- Screening MRI and 24 hour BP to be done only if echo LVH criteria fulfilled.***

#### APPENDIX 4 CONSORT

**(Boutron 2008) flow chart with projected numbers of participants throughout trial**.

**Screening**

All dialysis patients aged 18-80 screened for eligibility at dialysis clinics in NHS Tayside & NHS GGC (N=250,Tayside, N= 650 NHS GGC)

Analysed (n=34)
♦ Excluded from analysis (give reasons)(n= 4)

Analysed (n=34)
♦ Excluded from analysis (give reasons)(n= 4 )

**Analysis**

Review

Participants lost to follow-up (n=4)

*10% dropout or death rate over 1 year allowed for*

Allocated to placebo group

(n= 38)

- Baseline data collection

**1 Year Follow-Up**

Review

Participants lost to follow- (n=4)

*10% dropout or death rate over 1 year allowed for*

Allocated to allopurinol group (n=38)

- Baseline data collection

Refused consent

N= 50

Screen failures

N=38

♦  Exclusion criteria

Consented and randomised to study 114 approx

**Allocation**
